# Expanding the phenotypic and genotypic spectrum of *KCNT1*-related epilepsies

**DOI:** 10.1093/braincomms/fcag256

**Published:** 2026-07-08

**Authors:** Mathilde Gras, Gaelle Quentin-Romand, Nicole Chemaly, Giulia Barcia, KCNT1 consortium, Guido Rubboli, Rikke Steensbjerre Møller, Claudia Maria Bonardi, Mathieu Kuchenbuch, Marc Fitzgerald, David Bearden, Pin Fee Chong, Naomichi Matsumoto, Munetsugu Hara, Mitsuhiro Kato, Shin Nabatame, Kazuyuki Nakamura, Yushi Inoue, Jazmyn Karpathakis, Lucy Coulter, Rebekah Harris, Ingrid E Scheffer, Michael Hildrebrand, Romano Ferrucio, Valeria Capra, Renzo Guerrini, Simona Balestrini, Elena Parrini, Tsang H Y Mandy, Mak C Y Christopher, Fan S S Samuel, Chung H Y Brian, Francesca Ragona, Freri Elena, Jacopo C DiFrancesco, Barbara Castellotti, Tiziana Granata, Moisés Léon-Ruiz, Magdalena Krygier, Marta Zawadzka, Maria Mazurkiewicz-Bełdzińska, Liudmyla Turova, Khrystyna Shchubelka, Kaoru Yamamoto, Shimpei Baba, Azusa Ikeda, Audrey Oetomo, Douglas Nordli, Laura Licchetta, Francesca Bisulli, Juan Pablo Appendino, Karl Martin Klein, Ping-Yee Billie Au, Anita N Datta, Emily Spelbrink, Adam Numis, Marie-Coralie Cornet, Maria Roberta Cilio, Lara Adami, Marina Trivisano, Licia Salimbene, Silvia Tenembaum, Maria Cecilia Kravetz, Adeline L Vanderver, Amy Pizziano, Johanna Schmidt, Stéphane Auvin, Pierre Mayer, Rima Nabbout

**Affiliations:** Department of Pediatric Neurology, Reference Centre for Rare Epilepsies, Necker Enfants Malades Hospital, APHP, Member of EPICARE, Université Paris Cité, 149 rue de Sevres, 75015 Paris, France; Institut Imagine, INSERM U1163, Université Paris Cite, 24 boulevard Montparnasse, 75015 Paris, France; Department of Pediatric Neurology, Reference Centre for Rare Epilepsies, Necker Enfants Malades Hospital, APHP, Member of EPICARE, Université Paris Cité, 149 rue de Sevres, 75015 Paris, France; Institut Imagine, INSERM U1163, Université Paris Cite, 24 boulevard Montparnasse, 75015 Paris, France; Department of Pediatric Neurology, Reference Centre for Rare Epilepsies, Necker Enfants Malades Hospital, APHP, Member of EPICARE, Université Paris Cité, 149 rue de Sevres, 75015 Paris, France; Institut Imagine, INSERM U1163, Université Paris Cite, 24 boulevard Montparnasse, 75015 Paris, France; Department of Pediatric Neurology, Reference Centre for Rare Epilepsies, Necker Enfants Malades Hospital, APHP, Member of EPICARE, Université Paris Cité, 149 rue de Sevres, 75015 Paris, France; Institut Imagine, INSERM U1163, Université Paris Cite, 24 boulevard Montparnasse, 75015 Paris, France; Department of Pediatric Neurology, Reference Centre for Rare Epilepsies, Necker Enfants Malades Hospital, APHP, Member of EPICARE, Université Paris Cité, 149 rue de Sevres, 75015 Paris, France; Institut Imagine, INSERM U1163, Université Paris Cite, 24 boulevard Montparnasse, 75015 Paris, France

**Keywords:** KCNT1 gene, developmental and epileptic encephalopathy, epilepsy of infancy with migrating focal seizures, sleep-related hypermotor epilepsy, systemic-to-pulmonary collateral arteries

## Abstract

The *KCNT1* gene encodes for a sodium-activated potassium channel involved in neuronal excitability. Since its initial description in 2012 in patients with Epilepsy of Infancy with Migrating Focal Seizures (EIMFS) and in Sleep-related Hypermotor Epilepsy (SHE), the associated phenotypic spectrum has broadened—encompassing other focal epilepsies and Developmental and Epileptic Encephalopathies (DEEs)—and has included extra-neurological features. We aimed to characterize the neurological outcomes, extra-neurological features, mortality and genotype-phenotype correlations expanding the follow-up of the reported cases with *KCNT1* variants. A comprehensive literature review was performed to identify all reported cases of *KCNT1* pathogenic or likely pathogenic variants. Corresponding authors were contacted to obtain updated clinical data, including current vital status, epilepsy progression, extra-neurological features, cognitive and psychiatric status. The entire dataset, including updated data from the literature, was combined for subsequent analyses. A total of 316 patients from 88 publications were included. Follow-up data were obtained for 60 patients (from 28 papers and 11 countries), increasing the median age at last assessment from 4.4 to 6.0 years. 181 patients had an EIMFS phenotype, 62 had SHE, and 59 had various DEEs. Five individuals were asymptomatic parents, six had other focal epilepsies, and three had other phenotypes. Extra-neurological features were predominantly observed in patients with EIMFS and DEE, notably systemic-to-pulmonary collateral arteries, other vascular or cardiac malformations and various respiratory, orthopaedic or gastrointestinal disorders. The main cause of death was pulmonary complications (haemorrhage or infection). Genotype–phenotype correlations revealed a trend for variants in the first regulator of conductance of potassium (RCK1) domain to associate with EIMFS/non-EIMFS DEE, while SHE-associated variants were predominantly located in the second regulator of conductance of potassium (RCK2) domain. Additionally, variants p.Arg474Cys may confer increased risk for vascular malformations, but the issue appears to be broader, and systematic screening of patients carrying pathogenic *KCNT1* variants would allow us to better define this risk. This study offers a comprehensive understanding of the clinical spectrum and genotype-phenotype correlation in KCNT1-related disorders. This study has inherent biases related to the retrospective collection of already published data and underscores the need to develop multisource data methodologies and registries to reduce follow-up loss in real-world data collections based on health records.

## Teaser

Gras *et al*. detail genotypic and phenotypic data from 316 individuals with *KCNT1*-variants reported in literature, including updated clinical information for 60 of them. They expand the phenotypic spectrum, refine phenotype-genotype correlation, highlight extra-neurological—particularly vascular—manifestations, and confirm a trend toward RCK1- Epilepsy-of-Infancy-with-Migrating-Focal-Seizures/Developmental-Epileptic-Encephalopathy and RCK2- Sleep-related-Hypermotor-Epilepsy associations.

## Graphical Abstract

**Graphical Abstract:**
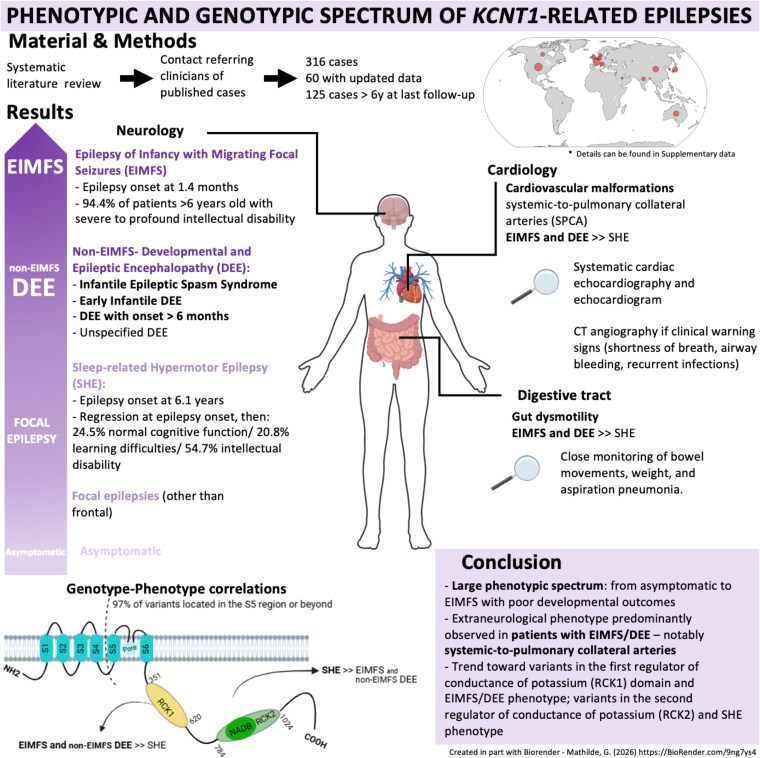

## Introduction

*KCNT1* gene encodes for a sodium-activated potassium channel, also known as Slack channel or Slo2.2.^1^ It is widely expressed in the nervous system, playing an important role in the regulation of the neuronal excitability,^2,3^ and it is also expressed in other organs like heart, kidney, testis,^1^ pancreas^4^ and proximal digestive tract.^5^ Its involvement in human pathology has been described since 2012, with reports of patients presenting with a phenotype of Early Infantile Migrating Focal Seizures (EIMFS)^6^ or a (Autosomal Dominant) Sleep-related Hypermotor Epilepsy phenotype (SHE).^7^ Since then, the neurological phenotypic spectrum has been refined and broadened with the description of various Developmental and Epileptic Encephalopathies (DEE) and other focal epilepsies.^8^ Additionally, pathogenic variants in *KCNT1* were reported in extra-neurological manifestations, such as Brugada syndrome^9^ and systemic-to-pulmonary collateral arteries (SPCA).^10^

The aim of our study was to review reported cases and series of individuals with *KCNT1* pathogenic and likely pathogenic variants and update their status at last follow-up in order to characterize the epilepsy syndrome, neurological outcome, the extra-neurological involvement as well as mortality and causes of death. To this end, we conducted an extensive comprehensive literature review and addressed a follow-up survey to all authors who reported these patients.

## Materials and methods

### Literature review

We performed a review of the literature in PubMed and EMBase using the keyword ‘KCNT1’ to identify all published cases with pathogenic and likely pathogenic *KCNT1* variants up to 28 February 2025. Search results were screened based on titles and abstracts, excluding those not written in English language, or those without patient data as experimental animal or *in vitro* studies. We excluded also systematic reviews based on published cases and not adding new ones. Potentially eligible articles were then reviewed in full. Three additional articles were identified through manual searches (by reviewing the references of the set of selected articles).

Among the selected articles, patients for whom clinical data were insufficient or for whom no molecular data was available were excluded. One patient was excluded due to an untraceable variant (incorrect protein coordinates). The ACMG criteria were used to assess the pathogenicity of the variants.

**Figure 1:**
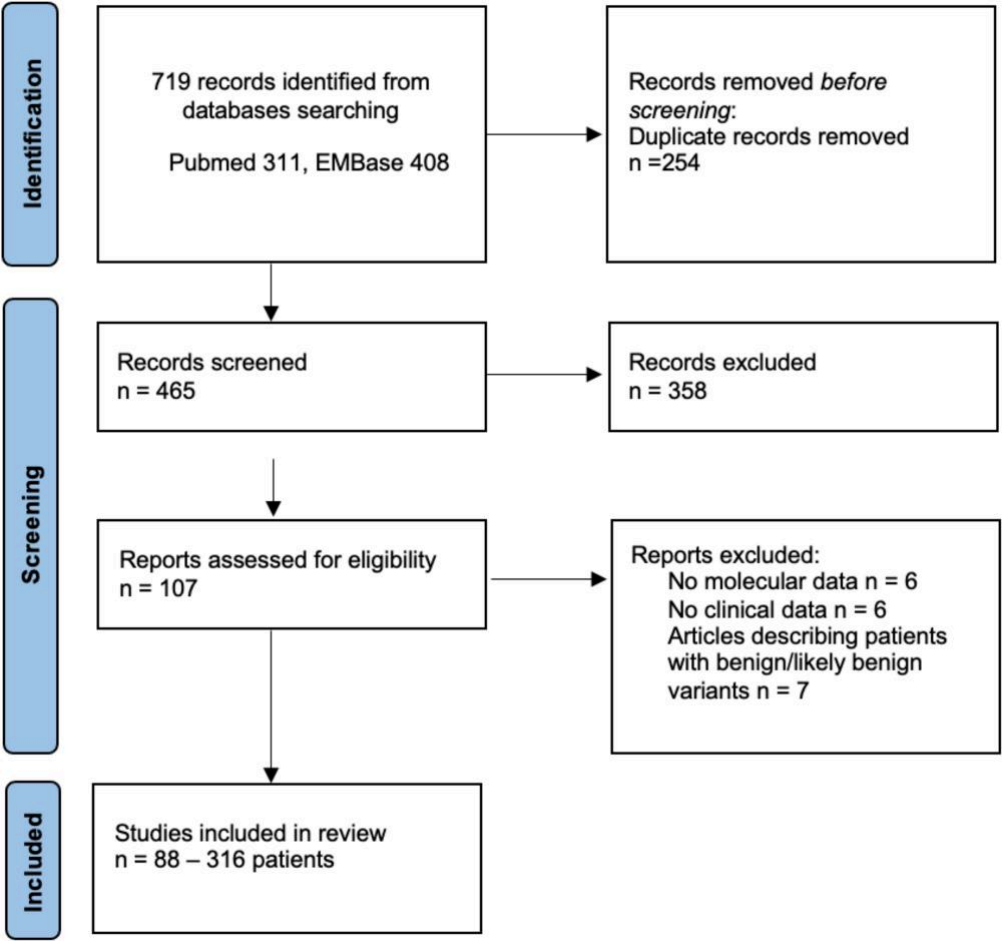
**Literature review flow chart.** A literature review was performed in PubMed and EMBase using the keyword ‘KCNT1’ to identify all papers reporting patients with pathogenic or likely pathogenic variants up to 28 February 2025. A total of 719 records were identified; 465 remained after duplicate removal and were screened. Of these, 358 were excluded (non-English language, no patient data, reviews without new cases, conference abstracts), and 107 full-text articles were assessed. After exclusion of studies without molecular or clinical data or reporting non-pathogenic or likely pathogenic variants, 88 articles were included.

In addition, patients harbouring other gene variants potentially interfering with the phenotype and patient with mosaic variants were also excluded. Search strategy and study selection are summarized in Fig. 1.

Following systematic cross-checking of demographic, genetic and clinical variables to minimize potential duplicate reporting across studies, relevant patient-level data were incorporated into the KCNT1 dataset.

The clinical and molecular data collected are available in the Supplementary material.

### Updating of patient outcomes

In order to extend the follow-up and to further explore neurological, extra-neurological outcomes and mortality, we contacted the corresponding authors of every included article by email, using the addresses provided in the published papers. Correspondent authors transferred the survey to their co-authors or advised us to do it directly searching for additional co-authors’ emails. We obtained a total of 215 email addresses for 84 articles, from 26 countries (Supplementary Fig. 1 and Supplementary Table 1).

**Figure 2:**
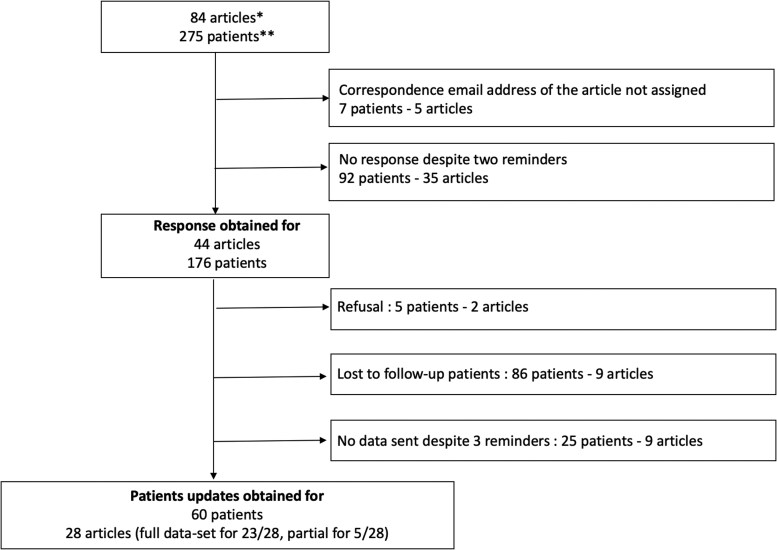
**Flowchart illustrating the process of retrieving follow-up information on previously published patients through renewed contact with the original reporting clinicians.** * Included articles were only articles reporting alive patients or with vital status unknown. Three articles were included in the literature review but were not included in the search for patient updates because they were identified too late (through manual searching). Burgess *et al*.^92^, Fang *et al*.^33^, Martin *et al*.^78^ were included in the literature review but not in the patient’s update. ** Alive or vital status unknown in the article.

Clinical update was requested through a survey on major clinical features at last follow-up, including patient’s age, neurological disorders (epilepsy, cognitive and motor functions and the results of updated brain imaging), as well as cardiovascular, gastrointestinal, orthopaedic, and psychiatric disorders. Response to treatment was not within the scope of the paper (Supplementary Table 2). The referent physicians that we identified with the correspondent authors completed the surveys. We sent monthly personal reminders based on the answers received with three reminders. The flow chart of this process is presented in Fig. 2.

Standardized assessments of psychomotor development were infrequently reported for the included studies. To harmonize cognitive outcome reporting across cohorts, cognitive status was categorized according to age. In children younger than 6 years, the term neurodevelopmental delay (NDD) was used, whereas in individuals aged 6 years or older, the term intellectual disability (ID) was applied.

For patients aged ≥6 years, mild ID was assigned when preserved language and partial age-appropriate autonomy were described; moderate ID when a significant delay with limited functional abilities was reported; and severe/profound ID in cases of absent language or explicitly described profound intellectual impairment. Because of the difficulty in reliably distinguishing between severe and profound ID in the absence of standardized developmental scales or formal psychometric testing, these categories were combined into a single ‘severe–profound ID’ group when classified as such by the referring clinicians.

In children younger than 6 years, NDD severity was determined based on the qualitative descriptions provided by the authors, taking into account functional abilities, communication skills, and developmental milestone attainment when available.

This study was approved by the Institutional Review Board (IRB) of APHP Centre, in accordance with the Declaration of Helsinki.

### Statistical analysis

GraphPad Prism was used to draw the figures and for statistical analyses. Comparison between group was done using Log-rank (Mantel Cox) test for survival analyses, Mann–Withney U-test for continuous non-parametric variables, Chi-squared test to compare distributions between groups of nominal categorical variables and Fisher’s exact test was used for dichotomous variables. Multiple comparisons were controlled using the false discovery rate (FDR) method according to Benjamini–Hochberg, with an adjusted *P*-value <0.05 considered statistically significant.

## Results

### Phenotypic features of *KCNT1* patients

**Figure 3:**
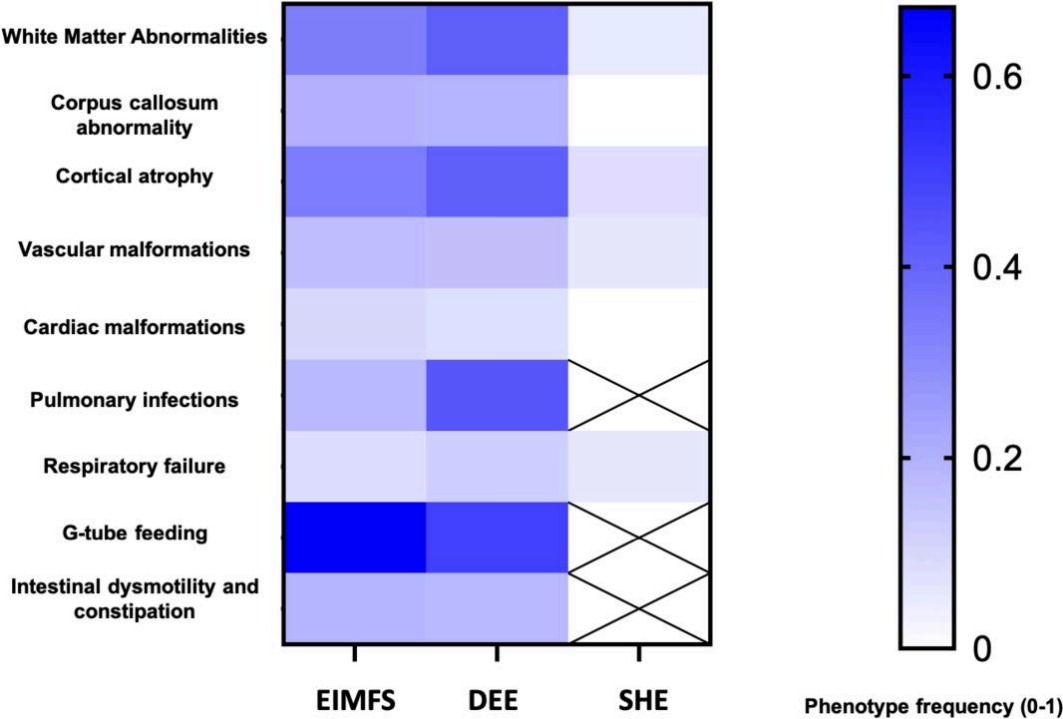
**Heat-map of the different neurological and extra-neurological features associated with *KCNT1* patients (EIMFS, DEE, SHE).** Colour intensity reflects the frequency of each feature within each group, with darker shades indicating higher prevalence. Crossed-out cells represent missing or unavailable data for the SHE group. Features include brain MRI abnormalities (data available for 141 EIMFS patients, 36 DEE patients, 38 SHE patients) and systemic involvement such as cardiovascular and/or respiratory problems (data available for 84 EIMFS patients, 25 DEE patients, 17 SHE patients) or gastrointestinal dysfunction (data available for 63 EIMFS patients, 22 DEE patients, 2 SHE patients). Fisher’s exact test was performed to compare the frequency of vascular malformations between the EIMFS and non-EIMFS DEE groups, without significant difference (Fisher’s exact test, *P* > 0.99). Vascular malformations were reported in only one patient with SHE, who had multiple small cavernomas. No additional statistical analysis was performed with these data. DEE, Developmental and Epileptic Encephalopathy; EIMFS, Epilepsy of Infancy with Migrating Focal Seizures; MRI, Magnetic Resonance Imaging; SHE, Sleep-related Hypermotor Epilepsy.

We analyzed clinical data from 316 individuals with *KCNT1* variants, compiled from 88 published studies.^3,6-93^ Follow-up clinical data were obtained directly for 60 individuals, reported across 28 articles (from 11 countries) (Supplementary Tables 3 and 4 and Supplementary Fig. 1). The main phenotypic features observed in this cohort are summarized in Tables 1 and 2 and Fig. 3.

**Table 1:** Neurological phenotypic features of the published patients (including updates of 60 patients)

	Phenotype			
Neurological phenotype features (*n*)	EIMFS [*n* = 181]	DEE [*n* = 59]	SHE [*n* = 62]	Other^a^ [*n* = 14]
Sex: Male (%)/Female (%)	91 (57.2%)/68 (42.8%) [*n* = 159]	33 (57.9%)/24 (42.1%) [*n* = 57]	39 (66.1%)/20 (33.9%) [*n* = 59]	7 (53.8%)/6 (46.2%) [*n* = 13]
Age: Median (min—max)	3.15 (0.17–24) [*n* = 146]	7 (0.33–37) [*n* = 47]	19 (4.75–60) [*n* = 36]	39.5 (15–64) [*n* = 8]
ND before seizure onset	[*n* = 64]	[*n* = 16]	[*n* = 28]	[*n* = 7]
Normal	56	11	25	7
Mild NDD	2	1	3	
Severe NDD	2	3		
NDD (severity unknown)	4	1		

Regression after seizure onset? Y/N	50/20 [*n* = 70]	15/10 [*n* = 25]	22/8 [*n* = 30]	4/0 [*n* = 4]
ND after seizure onset	[*n* = 158]	[*n* = 48]	[*n* = 53]	[*n* = 10]
Normal			13	6
Learning difficulties			11	1
Mild ID			14	3
Moderate NDD/ID	4	5	6	
Severe to profound NDD/ID	119	28	4	
NDD/ID (severity unknown)	35	15	5	
Brain MRI	[*n* = 141]	[*n* = 36]	[*n* = 38]	[*n* = 6]
Normal Brain MRI Y/*N*	66/75	13/23	31/7	5/1
White matter abnormality	47	15	2	
Including delayed myelinization	33	12	1	
Including white matter loss	3	1		
Without details	11	2	1	
Midline abnormality	28	7		
Including thin corpus callosum	26	6		
Cerebral atrophy	48	15	3	1
Other^b^	13	2	4	1

DEE, Developmental and Epileptic Encephalopathy; EIMFS, Epilepsy of Infancy with Migrating Focal Seizures; ID, Intellectual Disability; MRI, Magnetic Resonance Imaging; ND, NeuroDevelopmental; NDD, NeuroDevelopmental Delay; SHE, Sleep-related Hypermotor Epilepsy.

^a^Other phenotypes: asymptomatic (5), focal epilepsy other than frontal-lobe epilepsy (6), Brugada syndrome + seizures (1), Epilepsy with Myoclono-Atonic Seizures (1), Epilepsy with Tonic Clonic Seizures non-specified otherwise (1), Multifocal epilepsy (1).

^b^Other findings in brain MRI: For EIMFS: Abnormally open operculum (1), cerebellar atrophy (3), mild Chiari I (1), external hydrocephalus (1), dolichocephaly (2), hygromas (2), subdural hematoma (2), unilateral widening of sylvian fissure (1). For DEE: atrophy of cerebellar vermis (1), left ventricular dilatation (1). For SHE: cerebellar atrophy (2), L Periventricular nodular heterotopia with transmantle signs (1), multiple very small cerebral cavernomas with no sign of previous bleedings (1); Other: microbleed in medial pons (1).

**Table 2:** Extra-neurological phenotypic features of the published patients (including updates of 60 patients)

	Phenotype			
Extra-neurological phenotype features (*n*)	EIMFS [*n* = 181]	DEE [*n* = 59]	SHE [*n* = 62]	Other^a^ [*n* = 14]
Cardiovascular and/or respiratory problems Y/N	54/30 [*n* = 84]	17/8 [*n* = 25]	2/15 [*n* = 17]	1/3 [*n* = 4]
Vascular malformations (including SPCA)	14 (9)	4 (3)	1	
Cardiac malformations^b^	10	2		
Arrhythmia (included Brugada syndrome)	3		1	1 (1)
Aspiration/recurrent pneumonia	15	11		
Frequent upper respiratory tract infections	6			
Respiratory issues	7	4		
Respiratory failure/insufficiency	7	3	1	
Sleep apnoeas	3	1		
Orthopaedic problems (co-occurring)	[*n* = 31]	[*n* = 13]	[*n* = 1]	[*n* = 0]
Scoliosis	24	10	1	
Dysplasia or dislocation	16	5		
Osteopenia or fracture	4	2		
Other	9	6		
Gastrointestinal disorders	[*n* = 63]	[*n* = 22]	[*n* = 2]	[*n* = 0]
Oral alimentation	10	6		
Nasogastric-tube	4			
Gastrostomy	39	11	1	
GERD	12	4		
Constipation and intestinal dysmotility	12	4	1	
Endocrine disorders	[*n* = 6]	[*n* = 1]	[*n* = 3]	[*n* = 0]
Precocious puberty	5		3	
Hypothyroidism		1		
Neurosensory impairment Y/N	18/0 [*n* = 18]	6/1 [*n* = 7]		
Visual impairment	17	6		
Hearing impairment	1			
Deceased patients Y/N	33/124 [*n* = 157]	6/37 [*n* = 43]	3/54/[*n* = 57]	1/11 [*n* = 12]
Pulmonary haemorrhage	5	2		
Respiratory failure	3			
Respiratory infection	6			
Sudden Unexplained Death in Epilepsy	4	1	1	
Others^c^	7	2	1	
Undescribed aetiology	8	1	1	1

DEE, Developmental Epileptic Encephalopathy; EIMFS, Epilepsy of Infancy with Migrating Focal Seizures; GERD, Gastroesophageal Reflux Disease; SHE, Sleep-related Hypermotor Epilepsy; SPCA, Systemic-to-Pulmonary Collateral Arteries.

^a^Other phenotypes: asymptomatic (5), focal epilepsy other than frontal-lobe epilepsy (6), Brugada syndrome + seizures (1), Epilepsy with Myoclono-Atonic Seizures (1), Epilepsy with Tonic Clonic Seizures (1), Multifocal epilepsy (1).

^b^Cardiac malformations: For EIMFS: patent foramen ovale (1), ventricular septal defect (2), bicuspid aortic valve (1), dilatated cardiomyopathy (2), left ventricular hypertrophy (2), mitral regurgitation (2). For DEE: additional chord, metabolic cardiomyopathy (1), patent foramen ovale (1).

^c^Others causes of deaths: For EIMFS: Infections others than pneumonia (1), Stormy phase (2), Systemic illness (2), Disease progression and redirection of care (2). For DEE: Severe neurological illness (1), Extreme bradycardia (1). For SHE: Glioblastoma (1).

### Patients with EIMFS (*n* = 181)

Follow-up updates were obtained for 31 patients, allowing us to extend the median age at last follow-up from 2.5 years (range: 0.17–22; *n* = 145) to 3.15 years (range: 0.17–24; *n* = 146).

### Neurological phenotype

Among the 181 individuals with EIMFS, 57.2% were male. The mean age at seizure onset was 1.4 months [±1.7 Standard Deviation (SD), range 0.03–12 months]. Seizure frequency at onset was reported as daily or multiple times per day in 106/110 (96.4%) individuals. After the age of 6 years, seizures remained daily or multiple per day in 28/57 (49.1%). Three patients were reported as seizure-free (seizure freedom period of 2, 3 and 10 years) and four had sporadic seizures. Among the others, one had nightly seizures, six had weekly seizures, four had monthly seizures, and information was unavailable for 11 patients.

Psychomotor development following epilepsy onset was reported as delayed in all patients with available data (158/158). After age 6, 51/54 (94.4%) had developed a severe-profound ID and three were reported to have ID of unspecified severity. Among them, a notable case, Patient 17, previously described in 2019^45^ (p.Met896Ile), had an atypical developmental course. She experienced a seizure-free period between 9 and 32 months. During this interval, she acquired walking, speech, and social interaction skills, and at last follow-up at age 16 years, she remained verbal (able to form short sentences and answer simple questions), ambulatory, and attended a specialized institution. Nevertheless, due to the absence of a psychometric assessment, it was not possible to formally evaluate her level of ID (Supplementary Table 3).

Another notable case, Patient 1 reported in 2025^11^ (p.Arg398Gln), improved from severe developmental delay at 2.5 years to moderate delay by age 3. However, this last case has a very short follow-up and these results should be further confirmed.

Brain Magnetic Resonance Imaging (MRI) was performed for 141/181 patients. Median age when brain MRI performed was 4.5 months (range: from the first days of life to 10 years; age information unavailable for 67/141 patients). MRI was reported abnormal in 75/141 (53.2%) individuals, showing White Matter Abnormalities in 47 cases [including 33 Delayed Myelination (DM), 3 White Matter loss, and 11 WMA without details], thin Corpus Callosum in 26/75, and Cerebral Atrophy in 48/75 cases.

### Extra-neurological phenotype

Vascular malformations were reported in 14 cases and were evaluated or investigated in patients with pulmonary haemorrhage or other vascular symptoms. They included SPCA in nine cases^10,32,34,35,62,75,91^ and the following abnormalities (in one patient each): complex arteriovenous fistula between the hepatic artery and the left portal vein branch,^45^ ectasia of the bronchial and main pulmonary arteries,^57^ angiodysplasia of the bronchial arteries, abnormal pulmonary venous return and abdominal vascular anomalies,^11^ an aberrant right subclavian artery^92^ and a pulmonary angiodysplasia.^92^

Eight patients had structural cardiac anomalies (patent foramen ovale,^39^ ventricular septal defect,^39,92^ bicuspid aortic valve,^45^ dilatated cardiomyopathy,^45^ left ventricular hypertrophy,^10^ mitral regurgitation^45,75^).

In addition, 31 patients underwent an Electrocardiogram (ECG), which revealed arrhythmias in three individuals^39,45,75^ and conduction abnormalities—specifically prolonged QT interval—in two others.^35,46^

Recurrent or aspiration pneumonia was reported in 15 patients, and seven developed chronic respiratory insufficiency. Additional seven individuals showed unspecified respiratory disorders.

Gastro-intestinal disorders were informed for 63 patients. Enteral feeding was required in 43 of them (68.2%). Gastroesophageal Reflux Disease (GERD), gastrointestinal dysmotility or constipation were specifically reported in 24 (38.1%).

Orthopaedic issues were reported in 31 individuals. Scoliosis was the major symptom (24/31) followed by unilateral or bilateral hip dislocation or dysplasia in 16.

### Mortality

We extended the information on mortality up to 157 patients, with 33 reported deceased (21%). Vital status unknown for the remaining 24. Median age of death was 1.58 years (range: 0.2–19.5 years) with four patients who died at 1.5 years and 14 who died before the age of two. When reported, the most commonly reported causes of death were respiratory infections and insufficiency (9/33), pulmonary haemorrhage (5/33), and Sudden Unexpected Death in Epilepsy (SUDEP) (4/33).

### Patients with non-EIMFS-DEE (*n* = 59)

Non-EIMFS-DEE was identified in 59 patients. Follow-up updates were obtained for 15, allowing an increase in the median age at last examination from 4 years (range 0.33–31.5, *n* = 45) to 7 years (range 0.33–37, *n* = 47).

### Neurological phenotype

Individuals with non-EIMFS-DEE were categorized into three main groups:

- Infantile Epileptic Spasms Syndrome (IESS): 12 of them presented with epileptic spasms and a hypsarrhythmia EEG pattern,^8,26,67,75^ fulfilling criteria for IESS. Seizure evolution over time was not documented. Cognitive outcome was available for 4/12: one with ID level not precised, two with severe to profound ID, one with severe NDD (ages at last follow-up: 0.9, 1, 3 and 31 years). Brain MRI was performed in all four patients for whom cognitive data were available: Cortical Atrophy was reported in two (MRI at 7 and 20 months), callosotomy was reported in 1 (MRI at 5 months), and it was considered normal in the remaining case (MRI age not available).
- Early Infantile Developmental and Epileptic Encephalopathy (EIDEE): 28 patients fulfilled criteria for EIDEE.^8,16,34,39,44,48,52,54,55,71,73,78,80,91^ Initial seizures were predominantly focal in 9/12 individuals with available data. EEG at presentation showed burst-suppression in 9/17, multifocal discharges in 5/17, focal spikes in 1/17, generalized epileptic discharged in 1/17 and hypsarrythmia in 1/17. At last follow-up (median age 6.5 years, range 0.33–19), 16/28 continued to experience daily or multiple daily seizures. All presented with global developmental delay: two with unspecified ID and seven with NDD, two with moderate ID, nine with severe to profound ID and eight with severe NDD. Brain imaging was available for 24 individuals: six were reported as normal. Among the remaining 18, white matter abnormalities (WMA) were noted in 13 (delayed myelinization: 10; leukopathy: 1; non-specified WMA: 2), thin CC in 6, CA in 13, temporo-parieto-occipital dysplasia in 1, cerebellar vermis atrophy in 1, and unilateral ventricular dilatation in 1.
- Undetermined DEE: 5 individuals were reported with a DEE not further specified.^14,17,30^ Brain imaging was not performed. Regarding cognition, one had moderate NDD, one had severe NDD, one had severe to profound ID, and data were unavailable for the remaining two.
- Others: 14 individuals had an epilepsy onset later than EIDEE, with a median age at seizure onset of 6 months (range 4–36 months).^8,16,19,38,39,67^ Limited data were available on seizure and syndrome types and EEG findings. One was described as having Epilepsy with Myoclonic-Atonic Seizures (EMAtS) without further details.^49^ Over years, three individuals evolved toward a SHE-like phenotype.^8^ At last follow-up (median age 16 years, range 1.75–37), all presented with developmental delay: NDD in four, ID in two (unspecified level), moderate ID in one, and severe to profound ID in five. Brain MRI was available for eight: six were reported as normal and two showed DM.

### Extra-neurological phenotype

Four individuals (6.9%) presented with vascular malformations (including 3 SPCA). Seven of them were reported to have undergone an ECG, which identified a conduction abnormality (prolonged QT interval) in one case.^17^ 11/25 experienced aspiration/recurrent pneumonia, and three required ventilatory support. Gastrointestinal issues were frequent: 11/22 required enteral feeding, while 6/22 was able to feed orally without assistance. Constipation or intestinal dysmotility was reported in four individuals. Orthopaedic complications were also common: among the 13 individuals with available data, 10 had scoliosis and five had hip dislocation or dysplasia.

### Mortality

Including follow-up data from 15 individuals, six were deceased at a median age of 15 years (range 0.33–22.5 years old). Pulmonary haemorrhage was reported as the cause of death in two cases (Repeated massive bleeding from pulmonary plexiform haemangioma, in spite of repeated coil embolization in the past for one and secondary to SPCA).

### SHE (*n* = 62)

We obtained follow-up data for 10 individuals, extending the median follow-up duration from 17 years (range: 4.75–54, *n* = 34) to 19 years (range: 4.75–60, *n* = 36).

### Neurological phenotype

Sixty-two individuals were described with Autosomal Dominant SHE (ADSHE). Mean age at seizure onset was 6.1 years (±5 SD—range: 2 months—25 years), with predominantly frontal hypermotor seizures. Epilepsy was reported as drug-resistant in 31/47 individuals.

Neurodevelopment prior to seizure onset was reported in 28 individuals, described as normal in 25/28 and mildly delayed in 3/28. At last follow-up, 13/53 (24.5%) had normal cognitive function, 11/53 (20.8%) had learning difficulties, and 29/53 (54.7%) were described with ID (mild in 14, moderate ID in six, severe to profound ID or NDD in four, and unspecified level in five).

Brain MRI was performed in 38 individuals and reported as normal in 31. Median age when brain MRI performed was 9 years (range: from 5 months of life to 47 years; age information unavailable for 17 patients). Cortical atrophy was reported in three cases, associated in one with delayed myelination and cerebellar atrophy.^62^ White matter abnormalities, without further specification, were noted in one individual. One patient presented with small multiple cavernomas.^51^

Psychiatric comorbidities were also reported. Five individuals presented with hyperactivity, frequently associated with obsessive and aggressive behaviours, social difficulties, or Tourette syndrome. Autism spectrum disorder (ASD) was diagnosed in three patients. Two had Psychogenic Non-Epileptic Seizures (PNES). Three presented with mood, personality, or anxiety disorders requiring antidepressant treatment. Two individuals attempted suicide, and two reported suicidal ideation. Psychotic symptoms were described in three cases. One patient initially exhibited hyperkinetic and aggressive behaviour, followed by hypotonia and obtundation, with a catatonia-like presentation. Thirteen individuals exhibited unspecified psychiatric features, including behavioural disturbances and irritability. No psychiatric comorbidities were reported in five individuals (when specified).

### Extra-neurological phenotype

Regarding extra-neurological features, one patient presented with scoliosis, and three with precocious puberty. An ECG was performed in one patient, revealing an arrhythmia.^76^ None presented with vascular malformations.

### Mortality

Three patients had died, including one from SUDEP at 6 years old, one from glioblastoma at 49 years old, and one from unknown cause.^7,8^

### Other phenotypes (*n* = 14)

Five asymptomatic parents were reported: three within families with SHE phenotype,^8,53,76^ one mother of a patient with non-EIMFS-DEE,^39^ and one father of a patient with EIMFS.^62^

Three individuals were reported with focal temporal lobe epilepsy.^8,13^ The first entered epilepsy at age 8, developed pharmacoresistant seizures, severe depression, and mild cognitive regression. The second^8,13^ entered epilepsy at age 3, had pharmacoresistant seizures, and experienced marked improvement under fluoxetine treatment. At age 20, while treated with fluoxetine, clonazepam, and oxcarbazepine, he had been seizure-free for 2 years, with improvement in behaviour and mood, and had graduated from high school. The last one entered epilepsy at age 45, with focal dyscognitive seizures and bilateral convulsive seizures. To note, this last patient is harbouring the variant p.Arg133His which can be considered a VUS under the ACMG criteria; however, we chose to report it because it appears to be associated with this emerging phenotype.

Three patients were reported with focal epilepsy with presumed insular/opercular onset.^29^ One had pharmacoresistant seizures (frequent focal aware and impaired awareness seizures, rare focal to bilateral tonic-clonic seizures), while the other two had milder, pharmacosensitive epilepsy (one of whom was off treatment at the time of publication). All three belonged to a large family showing intrafamilial variability, including individuals with non-EIMFS-DEE, EIMFS and focal epilepsy.

Other patients could not be grouped: one had multifocal epilepsy and developed limbic encephalitis at seizure onset (anti-GAD65 antibodies), with persistent epilepsy and learning difficulties^76^; one was reported with both Brugada syndrome and epilepsy, with no additional information^9^; and one presented with generalized tonic-clonic seizures from age 3.8 years, mild cognitive regression, and pharmacosensitive epilepsy.^8^

### Inter-group comparisons

The age at epilepsy onset was correlated to the epilepsy syndrome with a significantly an earlier age of onset for patients with EIMFS compared with non-EIMFS DEE (Mann–Whitney test, *P* = 0.0014). Cognitive outcomes were statistically worst in EIMFS (Mann–Whitney test, *P* = 0.021), with only 4 of 123 patients (3.3%) exhibiting a moderate ID or NDD, compared with 5 of 33 (15.2%) in the non-EIMFS DEE group. In contrast, patients with SHE had a later age at epilepsy onset and a significantly more favourable cognitive prognosis, with 24 of 53 individuals (43%) showing no intellectual impairment (Mann–Whitney test, *P* < 0.0001).

Regarding the extra-neurological phenotype, no significant difference was observed in the frequency of vascular malformations between the EIMFS and non-EIMFS DEE groups (Fisher’s exact test, *P* > 0.99).

Of note, vascular malformations were identified in only one patient with SHE, who had multiple small cavernomas; no additional cases were reported in the SHE group.

**Figure 4:**
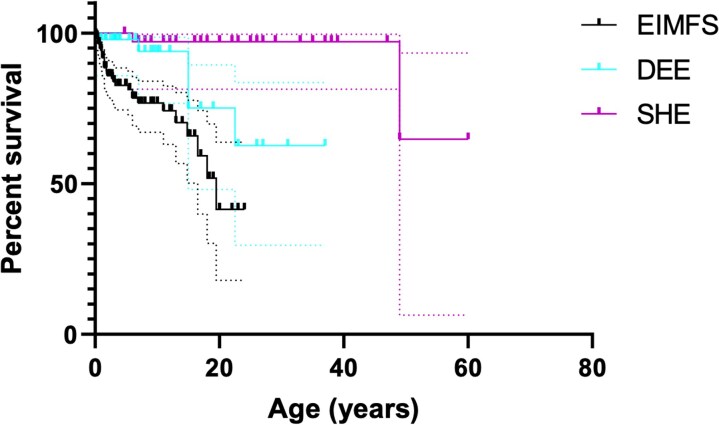
**Survival curves of patients with EIMFS (*n* = 157), DEE (*n* = 43) and SHE (*n* = 57), with 95% CI.** Comparison between group was done using Log-rank (Mantel Cox) test for survival analyses, showing reduced survival in the EIMFS group compared with both the non-EIMFS DEE group [Logrank (Mantel Cox)Test, *P* = 0.0188] and the SHE group [Logrank (Mantel Cox) Test, *P* < 0.0001]. DEE, Developmental and Epileptic Encephalopathy; EIMFS, Epilepsy of Infancy with Migrating Focal Seizures; SHE, Sleep-related Hypermotor Epilepsy.

Log-rank analysis demonstrated significantly reduced survival in the EIMFS group compared with both the non-EIMFS DEE group [Logrank (Mantel Cox) Test, *P* = 0.0188] and the SHE group [Logrank (Mantel Cox) Test, *P* < 0.0001]. In addition, survival was significantly lower in the non-EIMFS DEE group compared with the SHE group [Logrank (Mantel Cox) Test, *P* = 0.0109] (Fig. 4).

### Genotype–phenotype correlations

Inherited variants were significantly more frequent in the SHE group compared with both the EIMFS group (65.4% versus 6.2%; OR 28.7, 95% CI 12.2–69.3; *P* < 0.0001, Fisher’s exact test) and the non-EIMFS DEE group (65.4% versus 6.4%; OR 28.3, 95% CI 7.90–93.01; *P* < 0.0001, Fisher’s exact test).

**Figure 5:**
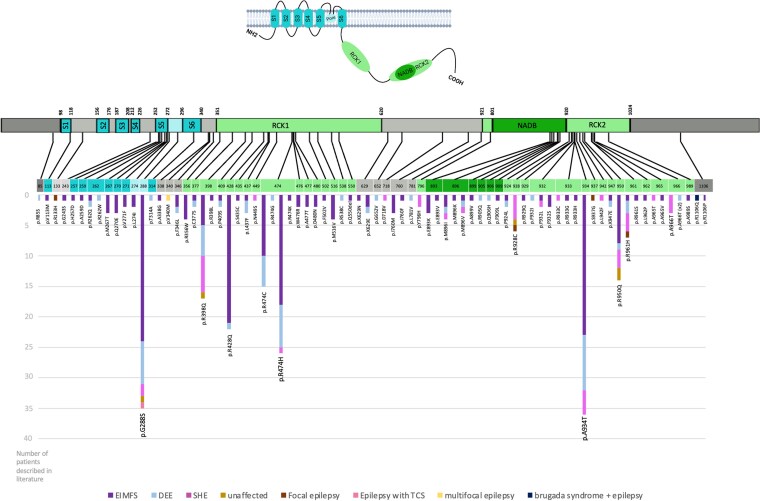
**Distribution of all KCNT1 variants reported in the literature along the protein, along with their associated phenotypes.** The number of patients is indicated on the *y*-axis of the figure. Each phenotype–family combination is represented once. The font size of the variants is representative of their frequency. DEE, Developmental Epileptic Encephalopathy; EIMFS, Epilepsy of Infancy with Migrating Focal Seizures; SHE, Sleep-related Hypermotor Epilepsy; TCS, Tonic-Clonic Seizures.

All reported variants and their associated phenotypes are presented in Fig. 5 and Supplementary Table 5. Notably, the majority of variants are located in the S5 region or beyond (283/287).

To determine whether specific protein domains were associated with distinct phenotypes, we compared phenotypes associated with variants located in the C-terminal, S5, pore, S6, RCK1, RCK2, NADBD, inter-RCK1–RCK2 regions, and other domains (Table 3). For the EIMFS and non-EIMFS DEE phenotype, we did not identify any significant difference in variant distribution across these major domains (*P* = 0.957, chi-squared test).

**Table 3:** Distribution of reported variants across major protein domains and their associated phenotypes

	EIMFS	DEE	SHE	Other	Total
**Nter**		1			1
**S5**	11	2			13
**pore**	26	7	2	2	37
**S6**	1	1		1	3
**RCK1**	76	24	8	1	109
**between RCK1—RCK2**	7	3	1		11
**RCK2**	44	16	23	6	89
**NADBD**	10	4	2		16
**Other domains**	4	1	0	1	6
**Cter**	1			1	2
**Total**	180	58	36	13	287

Each family (one phenotype) is counted once.

DEE, Developmental Epileptic Encephalopathy; EIMFS, Epilepsy of Infancy with Migrating Focal Seizures; SHE, Sleep-related Hypermotor Epilepsy.

Variants located within the RCK1 domain showed a trend toward association with EIMFS/non-EIMFS DEE (42% of variants in these phenotypes; OR 2.5, 95% CI 1.1–6.6; *P* = 0.028, FDR-adjusted *P* = 0.084, Fisher’s exact test), but did not reach statistical significance after correction for multiple comparisons. In contrast, variants located within the RCK2 domain were strongly associated with the SHE phenotype (64% of SHE-associated variants; OR 0.19, 95% CI 0.08–0.42; *P* < 0.0001, FDR-adjusted *P* < 0.0001, Fisher’s exact test). Given the number of comparisons, these results should be interpreted with caution.

Nevertheless, the observed associations between variants and specific protein domains were incomplete, indicating that these domains do not fully determine phenotype. A recurrent EIMFS-associated variant (p.Ala934Thr) was located in the RCK2 domain, and a recurrent SHE-associated variant (p.Arg398Gln) was found in the RCK1 domain. A list of recurrent variants is provided in Table 4. Variants were considered recurrent when reported more than three times.

**Table 4:** Recurrent variants

	EIMFS	DEE	SHE
p.Met267Thr	**3** (**1.7%)**		
p.Gln270Glu	**3** (**1.7%)**		
p.Gly288Ser	**24** (**13.3%)**	**7** (**11.8%)**	2 (5.6%)
p.Arg356Trp	**4** (**2.2%)**		
p.Arg398GIn	**5** (**2.8%)**	**5** (**8.5%)**	**6** (**16.7%)**
p.Arg428GIn	**21** (**11.7%)**	1 (1.7%)	
p.Arg474Cys	**10** (**5.6%)**	**5** (**8.5%)**	
p.Arg474His	**18** (**10%)**	**7** (**11.8%)**	1 (2.8%)
p.Met516Val	**4** (**2.2%)**		
p.Ile760Met	**3** (**1.7%)**		
p.Glu893Lys	**3** (**1.7%)**		
p.Arg928Cys			**4** (**11.1%)**
p.Ala934Thr	**23** (**12.8%)**	**9** (**15.2%)**	**4** (**11.1%)**
p.Arg950Gln	**8** (**4.4%)**	1 (1.7%)	**3** (**8.3%)**
p.Arg961His	1 (0.5%)	2 (3.4%)	**3** (**8.3%)**
p.Ala966Thr			**3** (**8.3%)**

Each family (one phenotype) is counted once. A variant was considered recurrent for a phenotype when three or more patients with that phenotype carried the variant (in bold).

DEE, Developmental Epileptic Encephalopathy; EIMFS, Epilepsy of Infancy with Migrating Focal Seizures; SHE, Sleep-related Hypermotor Epilepsy.

In addition, we report 15 families, with an overall putative penetrance of 87%. Among these families, 10 include patients with sleep-related hypermotor epilepsy (SHE), with a penetrance of 91%. Three families include a proband with epilepsy of infancy with migrating focal seizures (EIMFS) and an asymptomatic parent. One family comprises individuals with EIMFS and focal epilepsy, and one additional family includes individuals with focal epilepsy and an asymptomatic family member.

Notably, parental mosaicism was identified in seven other families, involving the father in two families and the mother in five.

**Figure 6:**
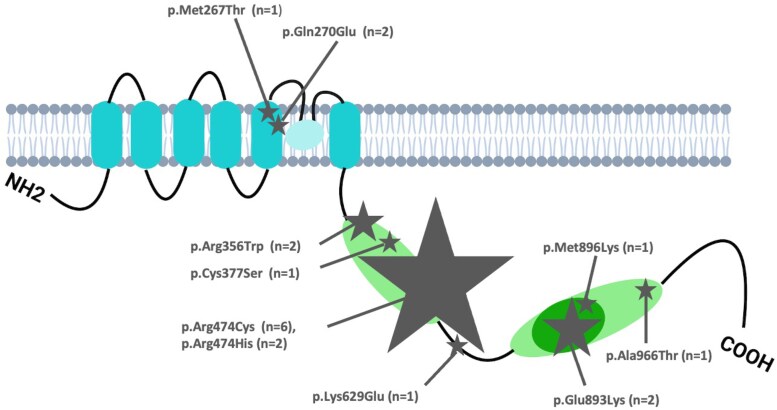
**Representation of KCNT1 variants associated with vascular malformations along the protein.** Each star represents the position of a variant for which at least one patient with a vascular anomaly has been reported. Larger stars indicate variants reported in a greater number of patients with vascular malformations. *n* indicates the number of reported cases for each corresponding variant. Created in BioRender. Mathilde, G. (2026) https://BioRender.com/d41ufm4.

We also explored a possible correlation between genotype and the presence of vascular malformations. A recurrent variant, p.Arg474Cys seems related to this endophenotype, although the issue should be considered in all patients with EIMFS and non-EIMFS DEE. Variants associated with vascular malformations are illustrated in Fig. 6.

It is interesting to note that the p.Arg474Cys variant was functionally studied (expression in Xenopus oocytes and HEK cell lines, and assessment of the variant’s impact on current magnitude, current–voltage relationships, and sodium ion modulation) by Hinckley *et al*.^94^ and appears to result in a reduction of function of the Slack channel. Variants reported to have undergone *in vivo* or *in silico* functional studies are listed in Supplementary Table 5. Three additional variants are reported as exhibiting loss of function: p.Gly652Val, p.Val340Met and p.Phe932Ile. No vascular abnormalities have been reported in patients carrying these variants; however, it is not specified whether this aspect was systematically investigated.

Among the loss-of-function variants, p.Phe932Ile is reported to be associated with a phenotype of epilepsy and marked cerebral hypomyelination. The cerebral myelination status of other patients with loss-of-function variants is difficult to assess: Among patients with the p.Arg474Cys variant, 8/15 show delayed myelination, but seven of these brain MRIs were performed before 12 months of age, with no subsequent follow-up imaging reported. No WMA were described in the patient carrying the p.Gly652Val variant (although the age at imaging is not available). No brain MRI is available for the patient with the p.Val340Met variant.

It is important to note that additional functional studies would certainly be necessary to confirm the loss-of-function nature of these variants: For example, the p.Tyr796His variant was reported as a loss-of-function variant by Hinckley *et al*.^94^ whereas other groups described it as a gain-of-function variant. These discrepancies underline that the interpretation of functional results should be approached with caution.

All other reported variants that have undergone functional studies are gain-of-function variants.

## Discussion

Our study investigated the phenotype and genotype of 316 patients with *KCNT1* variants with updated data for 60 of them. This allowed us to precise phenotypic spectrum with a long follow-up, to refine the phenotype-genotype correlation and to expand the information regarding neurological and extra-neurological phenotypes and on mortality.

### Epilepsy syndromes’ spectrum

Regarding the epilepsy phenotype, we delineated three epilepsy syndromes subgroups, EIMFS; SHE; and non-EIMFS DEE, expanding the latter specifically into EIDEE and IESS. Whereas the electroclinical delineation is straightforward between EIFMS and SHE as the age of onset, the seizures’ types and the cognitive outcomes are distinguishable, the boarders between EIFMS and EIDEE that initiates both in the first months of life with a severe epilepsy and neurologic outcome may be difficult to draw in some cases. The availability of long EEG recordings to identify the migrating pattern and the analysis of the suppression burst pattern in relation to therapies that may have induced it, may lead to some EIMFS patients being classified as EIDEE, thereby underestimating the spectrum of EIMFS phenotypes.

Regarding the brain imaging findings, WMA were reported in a number of patients with EIMFS/DEE. However, these findings should be interpreted with caution. Several factors may bias MRI interpretation, including the very young age at imaging, possible interpretation by non-pediatric neuroradiologists, incomplete information regarding concomitant treatments such as vigabatrin, and the limited availability of longitudinal neuroimaging data.

Regarding the extra-neurological phenotype, we observed that patients with early onset syndromes, both EIMFS and non-EIMFS DEE phenotypes are prone to cardiovascular malformations. Vascular malformations have been described, in particular SPCAs in patients with EIMFS or DEE. These collaterals can clinically manifest as heart failure, life-threatening haemoptysis, or pulmonary hypertension.^35^ Pulmonary haemorrhage is the second leading cause of death in these patients. However, the exact prevalence and natural history of systemic-to-pulmonary collaterals and other vascular malformations remain unknown due to the absence of systematic screening.

In addition to vascular anomalies, various structural cardiac anomalies have been also described as well as conduction disorders with QT prolongation^8,17,35,46^ and arrhythmias,^39,45,75,76^ including Brugada syndrome.^9,76^

Based on these findings, systematic cardiac echocardiography should be considered in all patients and CT angiography should be performed in patients presenting with cardiac dilation or dysfunction on screening echocardiogram, increasing shortness of breath or airway bleeding (haemoptysis or pulmonary haemorrhage), or recurrent infections.^95^ Such screening could help prevent cardiovascular complications and improve our understanding of the prevalence of this type of involvement among patients with *KCNT1*-related disorders.

On the respiratory side, patients with EIMFS and non-EIMFS DEE frequently presented with recurrent lower respiratory tract infections and respiratory failure. These are likely complications of the severe global disability observed in this population with different factors affecting the respiratory function: hypotonia and hypomotility, hypotonic respiratory muscular tone, scoliosis and gastro-intestinal reflux with possible micro or macro inhalation. Indeed, more than half of these patients required enteral feeding due to recurrent aspiration. Gastrointestinal symptoms such as gastro-intestinal dysmotility, gastro intestinal reflux and constipation were also common with a high percentage of tube feeding. More research should help to determine whether these disorders are directly related to the KCNT1 variant or secondary to the severe neurodevelopmental impairment. Their higher prevalence in individuals EIMFS and EIDEE compared with SHE and focal epilepsies supports at least a partial causal role of the severe neurodevelopment added to the direct impact of the variant.

### Genotype-phenotype correlation

In patients with EIMFS and those with non-EIMFS-DEE, variants were most commonly located in the RCK1 domains with no clear distinction in the variants’ location between both groups. By contrast, patients with SHE phenotypes more frequently carried variants in the RCK2 domain. However, these associations are not exclusive: EIMFS phenotypes were observed in patients with variants in the RCK2 domain, and vice versa. Even among recurrent variants, phenotypic variability was observed.

We also noted intrafamilial phenotypic variability, including families in which EIMFS, non-EIMFS DEE, and focal epilepsy with opercular/insular onset coexisted,^29^ or a father with autosomal dominant SHE (ADSHE) whose son had EIMFS,^83^ and families showing co-occurrence of EIMFS, non-EIMFS DEE, and SHE.^76^ In addition, five asymptomatic parents were identified: three had children with SHE, one with EIMFS, and one with non-EIMFS-DEE. No mosaicism was identified in the blood. This should be clearly stated for the genetic counseling. Among the families reported in the literature, we observed a putative penetrance of 87%. These results should be interpreted with caution, given the potential bias related to incomplete trio analyses (i.e. patient and both parents are not systematically tested) and the possible underestimation of certain mosaic cases due to the limitations of the genetic testing methodologies used. This variability may otherwise reflect the influence of genetic modifiers with the expansion of our knowledge on the polygenic risk factor in monogenic epilepsies^96^ but also on epigenetic mechanisms and environmental factors, which may modulate the functional consequences of *KCNT1* variants.

Intrafamilial variability and phenotypic variability of variants represent a major challenge for prediction, especially in the context of the expected rapid expansion of genomic newborn screening.^97-100^ In addition, the pressure of such prediction will increase with the initiation of antisense oligonucleotides trials^101^ and the expectation of a better efficacy with early therapies.^96^

### Mortality

We identified the main causes of death that varied in relation to the phenotypes: (i) respiratory infections and insufficiency and (ii) pulmonary haemorrhage reported in patients with EIMFS and DEE and (iii) SUDEP, which was reported across all three major groups (EIMFS, DEE, SHE). The cause of death was only reported in 74.4% of cases making difficult the definite calculation of SUDEP that was reported with considerable variability in EIMFS small series (ranging from 0%^39^ to 17%^45^).

Overall mortality rate was as high as 16.0%, with a significantly reduced survival in the EIMFS group compared with both the non-EIMFS DEE group and the SHE group. Survival was also significantly lower in the non-EIMFS DEE group compared with the SHE group. The unequal size of the subgroups may have introduced a sampling bias, potentially affecting the robustness of between-group comparisons. However, patients with DEEs with onset in infancy are at higher risk of mortality due to the severity of the disease beyond the SUDEP impact with some reports indicating that approximately half of children with DEE with onset in infancy die before the age of two.^102^ The mortality rate reported in our findings, including SUDEP, may be underestimated because of the missing follow-up data and because some of the loss of follow-up can be due to patient death.

### Tracking follow-up of published patients

Our study showed the difficulty of tracking published cases with 19.0% of patients having their data updated. This result may seem modest but should be analysed in the context of series reports with a large number of authors and a crossectional collection of data. This difficulty may be explained by various reasons: The changes of institutions by the physicians, the changes of addresses of the families, the transitory scientific interest based on an original finding as response to medication that decreases over time, the transitory nature of funds in many of the clinical research projects with the follow-ups often ending with the publication of the paper, the lack of registries, of electronic health records mining systems or time or human resources in some centres to go back to the files and to retrieve the data.

Optimizing the exploitation of electronic health records using data mining AI tools, the development of registries, may be part of the alternative to facilitate such studies.^103^

Similar studies should also face the risk of duplicates as there are no registries incorporating international pseudonymized identifiers. Although this risk was minimized in this study through systematic cross-checking potential duplicate cases, we cannot completely exclude this problem. The implementation of harmonized infrastructures would help to reduce the likelihood of artificial overestimation of specific phenotype's frequencies.

The involvement of families and patients advocacy groups as guarantors for these data collection is another possibility to keep on data updating. Developing and funding registries can have a key role. Registries when available are viewed as a powerful tool for developing clinical research and inform on natural history. The application of FAIR—Findable, Accessible, Interoperable, Reusable—principles is fundamental to enhance the impact and effectiveness of data derived from rare disease registries and enabling international data collection.^104^ Our ongoing project on K-channelopathies and the collaboration with KCNT1 advocacy groups and other K-related epilepsies groups will support further research on KCNT1 (https://epilepsies-innov4epik.com/fr/project/).

### Limitations

This study has some limitations related to its nature and originality. The retrospective nature of data collection and the heterogeneity of data reported in the literature introduce multiple potential biases. We attempted to mitigate the heterogeneity of the published data by contacting the clinicians who originally reported the cases in order to expand the data collection and to update it. We were able to increase the follow-up duration in the cohorts reported, but the low response rate (19.0%) may have introduced an additional bias.

Indeed, the combined approach introduces heterogeneity in the data sources, and this heterogeneity may have introduced selection bias. Importantly, we could not determine whether some of the loss of follow-up was due to patient death, or a loss of contact with the referral centre that published the case, or a discontinuation of hospital follow-up due to the patient’s clinical stability. This leads to risks of survivorship bias and selective reporting of complex cases.

The lack of uniform scales and in many cases of detailed standardized scales prevented us from reporting detailed and granular assessment of the cognitive functions’ disorders. This is inherent to the lack of such scales in the population of DEEs and to the availability these data mainly based on clinical evaluation.

## Supplementary Material

fcag256_Supplementary_Data

## Data Availability

The data supporting the findings of this study are available on request from the corresponding author.

## Appendix 1

### KCNT1 consortium

Guido Rubboli,^1,2^ Rikke Steensbjerre Møller,^1^ Claudia Maria Bonardi,^1,3^ Mathieu Kuchenbuch,^4^ Marc Fitzgerald,^5^ David Bearden,^6^ Pin Fee Chong,^7^ Naomichi Matsumoto,^8,9,10^ Munetsugu Hara,^11^ Mitsuhiro Kato,^12^ Shin Nabatame,^13,14^ Kazuyuki Nakamura,^15^ Yushi Inoue,^16^ Jazmyn Karpathakis,^17^ Lucy Coulter,^17^ Rebekah Harris,^17^ Ingrid E. Scheffer,^17,18,19^ Michael Hildrebrand,^17,20^ Romano Ferrucio,^21^ Valeria Capra,^21^ Renzo Guerrini,^22,23,24^ Simona Balestrini,^22,23^ Elena Parrini,^22^ Tsang HY Mandy,^25^ Mak CY Christopher,^25^ Fan SS Samuel,^25^ Chung HY Brian,^25^ Francesca Ragona,^26^ Freri Elena,^26^ Jacopo C. DiFrancesco,^27^ Barbara Castellotti,^28^ Tiziana Granata,^26^ Moisés Léon-Ruiz,^29^ Magdalena Krygier,^30^ Marta Zawadzka,^30^ Maria Mazurkiewicz-Bełdzińska,^30^ Liudmyla Turova,^31^ Khrystyna Shchubelka,^32,33^ Kaoru Yamamoto,^34^ Shimpei Baba,^34^ Azusa Ikeda,^35^ Audrey Oetomo,^36^ Douglas Nordli,^36^ Laura Licchetta,^37^ Francesca Bisulli,^37,38^ Juan Pablo Appendino,^39,40,41^ Karl Martin Klein,^42^ Ping-Yee Billie Au,^43,44^ Anita N. Datta,^45^ Emily Spelbrink,^46^ Adam Numis,^47^ Marie-Coralie Cornet,^48^ Maria Roberta Cilio,^49^ Lara Adami,^50^ Marina Trivisano,^51^ Licia Salimbene,^51^ Silvia Tenembaum,^52^ Maria Cecilia Kravetz,^53^ Adeline L Vanderver,^54,55^ Amy Pizziano,^54^ Johanna Schmidt,^54^ Stéphane Auvin,^56,57,58,59^ Pierre Mayer^60^

Affiliations:

1- Department of Epilepsy Genetics and Personalized Treatment, Member of ERN Epicare Network, Danish Epilepsy Centre, Dianalund, Denmark

2- University of Copenaghen, Copenaghen, Denmark

3- Pediatric Intensive Care Unit, Department of Woman’s and Child’s Health, University Hospital of Padova, Padova, Italy

4- Université de Lorraine, CHRU-Nancy, Service de Pédiatrie, Reference centre for rare epilepsies, member of ERN EpiCARE, Nancy, France

5- Division of Neurology, Departments of Neurology and Pediatrics. The Children’s Hospital of Philadelphia and the Perelman School of Medicine at the University of Pennsylvania, 3501 Civic Centre Blvd, Philadelphia, PA 19104, USA

6- Division of Child Neurology, Department of Neurology, University of Rochester School of Medicine, Rochester, NY, USA

7- Department of Pediatrics, Graduate School of Medical Sciences, Kyushu University, Fukuoka, Japan

8- Department of Human Genetics, Yokohama City University Graduate School of Medicine, Yokohama, Japan

9- Departments of Rare Disease Genomics and Clinical Genetics, Yokohama City University Hospital, Yokohama, Japan

10- Medical Genome Centre, National Centre of Neurology and Psychiatry, Kodaira, Japan

11- Department of Pediatrics and Child, Health, and Cognitive and Molecular Research Institute of Brain Disease, Kurume University School of Medicine, Kurume, Japan

12- Department of Pediatrics, Showa Medical University School of Medicine, Epilepsy Medical Centre, Showa Medical University Hospital, Tokyo, Japan

13- Department of Pediatrics, National Hospital Organization, National Osaka Hospital, Osaka, Japan

14- Department of Pediatrics, Graduate School of Medicine, the University of Osaka, Suita, Japan

15- Department of Pediatrics, Faculty of Medicine, Yamagata University, Japan

16- NHO Shizuoka Institute of Epilepsy and Neurological Disorders, Shizuoka, Japan

17- Epilepsy Research Centre, Department of Medicine, University of Melbourne, Austin Health, Heidelberg, Victoria, Australia

18- Department of Paediatrics, University of Melbourne, Royal Children’s Hospital, Melbourne, Australia

19- Florey Institute and Murdoch Children’s Research Institute, Melbourne, Australia

20- Neuroscience Group, Murdoch Children’s Research Institute, The Royal Children’s Hospital, Parkville, Victoria, Australia

21- Genomics and Clinical Genetics Unit, IRCCS Istituto Giannina Gaslini, Genoa, Italy

22- Neuroscience and Medical Genetics Department, Meyer Children's Hospital IRCCS, Florence, Italy

23- Department of NEUROFARBA, University of Florence, Florence, Italy

24- Neuroscience and Medical Genetics Department, Meyer Children's Hospital IRCCS, Florence, Italy

25- Department of Paediatrics and Adolescent Medicine, School of Clinical Medicine, LKS Faculty of Medicine, The University of Hong Kong, HK China

26- Department of Pediatric Neuroscience, member of the European Reference Network EPIcare, Fondazione IRCCS Istituto Neurologico Carlo Besta, Milan, Italy

27- Department of Neurology, Fondazione IRCCS San Gerardo Dei Tintori, Monza, Italy

28- Unit of Medical Genetics and Neurogenetics, Fondazione IRCCS Istituto Neurologico Carlo Besta, Milan, Italy

29- Section of Clinical Neurophysiology, Department of Neurology, La Paz University Hospital, Madrid, Spain

30- Department of Developmental Neurology Medical University of Gdańsk, Poland

31- Bogomolets National Medical university, Kyiv, Ukraine

32- Department of Biological Sciences, Oakland University, 118 Library Drive, Rochester, MI 48309, USA 33- Department of Biology, State University ‘Uzhhorod National University’, Voloshyna street, 32, Uzhhorod 88000, Ukraine

34- Department of Pediatric Neurology National Centre Hospital, National Centre of Neurology and Psychiatry, Tokyo, Japan

35- Department of Neurology, Kanagawa Children’s Medical Centre, Japan

36- Section of Child Neurology, Department of Pediatrics, Pritzker School of Medicine of the University of Chicago, Chicago, IL, USA

37- IRCCS Istituto delle Scienze Neurologiche di Bologna, Full Member of the European Reference Network for Rare and Complex Epilepsies (EpiCARE), Bologna, Italy

38- Department of Biomedical and NeuroMotor Sciences, Alma Mater Studiorum—University of Bologna, Bologna, Italy

39- Adjunct Professor. Department of Pediatrics, Cumming School of Medicine, University of Calgary, Calgary, Alberta, Canada

40- Professor. Department of Pediatrics, College of Medicine, University of Saskatchewan, Saskatoon, Saskatchewan, Canada

41- Pediatric Neurology Division, Department of Pediatrics, Jim Pattison Children's Hospital, Saskatoon, Saskatchewan, Canada

42- Departments of Clinical Neurosciences, Medical Genetics and Community Health Sciences, Hotchkiss Brain Institute & Alberta Children's Hospital Research Institute, Cumming School of Medicine, University of Calgary

43- Department of Medical Genetics, Cumming School of Medicine, University of Calgary, Calgary, Alberta, Canada.

44- Department of Pediatrics, University of Calgary, Alberta Children's Hospital, Calgary, Alberta, Canada

45- Clinical Associate Professor of Pediatrics (Neurology) University of British Columbia BC Children's Hospital Vancouver, Canada

46- Department of Neurology, Division of Child Neurology, Stanford Children's Hospital, Stanford University, Palo Alto, California, USA

47- Associate Professor of Neurology & Pediatrics, University of California, San Francisco, UCSF Benioff Children's Hospitals

48- Department of Pediatrics, UCSF Benioff Children's Hospital, University of California, San Francisco

49- Division of Pediatric Neurology, Cliniques Universitaires Saint-Luc, Université catholique de Louvain, Brussels

50- Department of Biomedical and Clinical Science, University of Milan, Milan, Italy

51- Neurology, Epilepsy and Movement Disorders Unit Bambino Gesù Children's Hospital, IRCCS, Rome, Italy

52- Department of Neurology, Pediatric Hospital Dr. J. Garrahan, Buenos Aires, Argentina

53- Department of Pharmacology, Faculty of Farmacy and Biochemistry, University of Buenos Aires, Buenos Aires City, Argentina

54- Division of Neurology, Department of Pediatrics, Children Hospital of Philadelphia

55- Department of Neurology, Perelman School of Medicine, University of Pennsylvania

56- AP-HP, Pediatric Neurology Department, Reference Centre for Rare Epilepsies, Member of ERN Epicare, Hôpital Universitaire Robert Debré, Paris, France

57- Institut Hospitalo-Universitaire Robert-Debré du Cerveau de l’Enfant, Paris, France

58- Université Paris-Cité, INSERM NeuroDiderot, Paris, France

59- Institut Universitaire de France (IUF), Paris, France

60- Neuropédiatrie, CHRU Montpellier, PhyMedExp, CNRS, INSERM, Université de Montpellier, France
