# Supplementary material for: Expanding the phenotypic and genotypic spectrum of KCNT1-related epilepsies
Source: Brain Commun. 2026 Jul 8;8(4):fcag256. doi: 10.1093/braincomms/fcag256 (PMC13367327; doi:10.1093/braincomms/fcag256)
Supplement: fcag256_Supplementary_Data [file fcag256_supplementary_data.pdf]

Supplementary data :

KCNT1 consortium, list of authors :

Guido Rubboli[1,2], Rikke Steensbjerre Møller[1], Claudia Maria Bonardi[1,3], Mathieu Kuchenbuch[4], Marc Fitzgerald[5], David Bearden[6], Pin Fee Chong[7], Naomichi Matsumoto[8,9,10], Munetsugu Hara[11], Mitsuhiro Kato[12], Shin Nabatame[13,14], Kazuyuki Nakamura[15], Yushi Inoue[16], Jazmyn Karpathakis[17], Lucy Coulter[17], Rebekah Harris[17], Ingrid E. Scheffer[17,18,19], Michael Hildrebrand[17,20], Romano Ferrucio[21], Valeria Capra[21], Renzo Guerrini[22,23,24], Simona Balestrini[22,23], Elena Parrini[22], Tsang HY Mandy[25], Mak CY Christopher[25], Fan SS Samuel[25], Chung HY Brian[25], Francesca Ragona[26], Freri Elena[26], Jacopo C. DiFrancesco[27], Barbara Castellotti[28], Tiziana Granata[26], Moisés Léon-Ruiz[29], Magdalena Krygier[30], Marta Zawadzka[30], Maria Mazurkiewicz-Bełdzińska[30], Liudmyla Turova[31], Khrystyna Shchubelka[32,33], Kaoru Yamamoto[34], Shimpei Baba[34], Azusa Ikeda[35], Audrey Oetomo[36], Douglas Nordli[36], Laura Licchetta[37], Francesca Bisulli[37,38], Juan Pablo Appendino [39,40,41], Karl Martin Klein[42], Ping-Yee Billie Au[43,44], Anita N. Datta[45], Emily Spelbrink[46], Adam Numis[47], Marie-Coralie Cornet[48], Maria Roberta Cilio[49], Lara Adami[50], Marina Trivisano[51], Licia Salimbene[51], Silvia Tenembaum[52], Maria Cecilia Kravetz[53], Adeline L Vanderver[54,55], Amy Pizziano[54], Johanna Schmidt[54], Stéphane Auvin[56,57,58,59], Pierre Mayer[60]

Affiliations:

1- Department of Epilepsy Genetics and Personalized Treatment, Member of ERN Epicare Network, Danish Epilepsy Center, Dianalund, Denmark;

2- University of Copenaghen, Copenaghen, Denmark

3- Pediatric Intensive Care Unit, Department of Woman's and Child's Health, University Hospital of Padova, Padova, Italy

4- Université de Lorraine, CHRU-Nancy, Service de Pédiatrie, Reference centre for rare epilepsies, member of ERN EpiCARE, Nancy, France.

5- Division of Neurology, Departments of Neurology and Pediatrics. The Children's Hospital of Philadelphia and the Perelman School of Medicine at the University of Pennsylvania, 3501 Civic Center Blvd, Philadelphia, PA 19104, USA

6- Division of Child Neurology, Department of Neurology, University of Rochester School of Medicine, Rochester, NY, USA

7- Department of Pediatrics, Graduate School of Medical Sciences, Kyushu University, Fukuoka, Japan.

8- Department of Human Genetics, Yokohama City University Graduate School of Medicine, Yokohama, Japan

9- Departments of Rare Disease Genomics and Clinical Genetics, Yokohama City University Hospital, Yokohama, Japan

10- Medical Genome Center, National Center of Neurology and Psychiatry, Kodaira, Japan

11- Department of Pediatrics and Child, Health,and Cognitive and Molecular Research Institute of Brain Disease, Kurume University School of Medicine, Kurume, Japan

12- Department of Pediatrics, Showa Medical University School of Medicine, Epilepsy Medical Center, Showa Medical University Hospital, Tokyo, Japan

13- Department of Pediatrics, National Hospital Organization, National Osaka Hospital, Osaka, Japan

14- Department of Pediatrics, Graduate School of Medicine, the University of Osaka, Suita, Japan

15- Department of Pediatrics, Faculty of Medicine, Yamagata University, Japan

16- NHO Shizuoka Institute of Epilepsy and Neurological Disorders, Shizuoka, Japan

17- Epilepsy Research Centre, Department of Medicine, University of Melbourne, Austin Health, Heidelberg, Victoria, Australia

18- Department of Paediatrics, University of Melbourne, Royal Children’s Hospital, Melbourne, Australia

19- Florey Institute and Murdoch Children’s Research Institute, Melbourne, Australia

20- Neuroscience Group, Murdoch Children’s Research Institute, The Royal Children’s Hospital, Parkville, Victoria, Australia

21- Genomics and Clinical Genetics Unit, IRCCS Istituto Giannina Gaslini, Genoa, Italy.

22- Neuroscience and Medical Genetics Department, Meyer Children's Hospital IRCCS, Florence, Italy;

23- Department of NEUROFARBA, University of Florence, Florence, Italy.

24- Neuroscience and Medical Genetics Department, Meyer Children's Hospital IRCCS, Florence, Italy.

25- Department of Paediatrics and Adolescent Medicine, School of Clinical Medicine, LKS Faculty of Medicine, The University of Hong Kong, HK China

26- Department of Pediatric Neuroscience, member of the European Reference Network EPIcare, Fondazione IRCCS Istituto Neurologico Carlo Besta, Milan, Italy.

27- Department of Neurology, Fondazione IRCCS San Gerardo Dei Tintori, Monza, Italy.

28- Unit of Medical Genetics and Neurogenetics, Fondazione IRCCS Istituto Neurologico Carlo Besta, Milan, Italy.

29- Section of Clinical Neurophysiology, Department of Neurology, La Paz University Hospital, Madrid, Spain.

30- Department of Developmental Neurology Medical University of Gdańsk, Poland

31- Bogomolets National Medical university, Kyiv, Ukraine.

32- Department of Biological Sciences, Oakland University, 118 Library Drive, Rochester, MI 48309, USA 33- Department of Biology, State University "Uzhhorod National University", Voloshyna street, 32, Uzhhorod 88000, Ukraine

34- Department of Pediatric Neurology National Center Hospital, National Center of Neurology and Psychiatry, Tokyo, Japan.

35- Department of Neurology, Kanagawa Children's Medical Center, Japan

36- Section of Child Neurology, Department of Pediatrics, Pritzker School of Medicine of the University of Chicago, Chicago, IL, USA

37- IRCCS Istituto delle Scienze Neurologiche di Bologna, Full Member of the European Reference Network for Rare and Complex Epilepsies (EpiCARE), Bologna, Italy.

38- Department of Biomedical and NeuroMotor Sciences, Alma Mater Studiorum – University of Bologna, Bologna, Italy

39- Adjunct Professor. Department of Pediatrics, Cumming School of Medicine, University of Calgary, Calgary, Alberta, Canada.

40- Professor. Department of Pediatrics, College of Medicine, University of Saskatchewan, Saskatoon, Saskatchewan, Canada.

41- Pediatric Neurology Division, Department of Pediatrics, Jim Pattison Children's Hospital, Saskatoon, Saskatchewan, Canada.

42- Departments of Clinical Neurosciences, Medical Genetics and Community Health Sciences, Hotchkiss Brain Institute & Alberta Children's Hospital Research Institute, Cumming School of Medicine, University of Calgary

43- Department of Medical Genetics, Cumming School of Medicine, University of Calgary, Calgary, Alberta, Canada.

44- Department of Pediatrics, University of Calgary, Alberta Children's Hospital, Calgary, Alberta, Canada

45- Clinical Associate Professor of Pediatrics (Neurology) University of British Columbia BC Children's Hospital Vancouver, Canada

46- Department of Neurology, Division of Child Neurology, Stanford Children's Hospital, Stanford University, Palo Alto, California, U.S.A.

47- Associate Professor of Neurology & Pediatrics

University of California, San Francisco

UCSF Benioff Children's Hospitals

48- Department of Pediatrics, UCSF Benioff Children's Hospital, University of California, San Francisco.

49- Division of Pediatric Neurology, Cliniques Universitaires Saint-Luc, Université catholique de Louvain, Brussels

50- Department of Biomedical and Clinical Science, University of Milan, Milan, Italy

51- Neurology, Epilepsy and Movement Disorders Unit Bambino Gesù Children's Hospital, IRCCS, Rome, Italy

52- Department of Neurology, Pediatric Hospital Dr. J. Garrahan, Buenos Aires, Argentina

53- Department of Pharmacology, Faculty of Farmacy and Biochemistry, University of Buenos Aires, Buenos Aires City, Argentina

54- Division of Neurology, Department of Pediatrics, Children Hospital of Philadelphia.

55- Department of Neurology, Perelman School of Medicine, University of Pennsylvania

56- AP-HP, Pediatric Neurology Department, Reference Center for Rare Epilepsies, Member of ERN Epicare, Hôpital Universitaire Robert Debré, Paris, France

57- Institut Hospitalo-Universitaire Robert-Debré du Cerveau de l'Enfant, Paris, France

58- Université Paris-Cité, INSERM NeuroDiderot, Paris, France

59- Institut Universitaire de France (IUF), Paris, France

60- Neuropédiatrie, CHRU Montpellier, PhyMedExp, CNRS, INSERM, Université de Montpellier, France

	Non responder	Partial responder	Responder
**Year of paper publication**			
< 2015	12 (86%)	1 (7%)	1 (7%)
2015-2020	27 (70%)	3 (7%)	9 (23%)
2020-2025	21 (60%)	1 (3%)	13 (37%)
**Continent**			
Europe	18 (60%)	2 (7%)	10 (33%)
Asia	24 (75%)	1 (3%)	7 (22%)
North America	14 (74%)	0	5 (26%)
Souht America	0	0	1
Australia	3 (60%)	2 (40%)	0
Africa	1	0	0
**Type of pathology reported**			
Early-onset epilepsy	43 (67%)	1 (1%)	20 (32%)
Late-onset epilepsy	9 (64%)	3 (22%)	2 (14%)
Both	8 (80%)	1 (10%)	1 (10%)
**Team with expertise in the team (> 1 publication on KCNT1)**			
Yes	24 (65%)	3 (8%)	10 (27%)
No	36 (70%)	2 (4%)	13 (26%)

Supplementary Table 1: Comparison of demographics of responders vs. non-responders

A

Paper reference (ex: Nabbout et al. 2024)
Patient identification in your paper (ex: patient 1) - *please use the same anonymisation that you used for the article*
Age at last visit (year)
Death since the publication (Y/N)
If yes, age at death (years)
If yes, cause of death
Head circumference (OFC) at last visit (SD)
Active epilepsy at last follow-up (> 1 seizure/year) (Y/N)
If yes, add frequency
Seizure types at last follow-up (generalized/focal/both/unknown)
ASM at last follow-up? (Y/N)
If yes, please detail them (ex: sodium valproate, lamotrigine, Cannabidiol…)
KD at last follow-up? (Y/N)
Epilepsy surgery since the publication ? (Y/N)
If yes, age at surgery
Type of surgery (resection/VNS…)
Feeding disorders (Y/N)
G tube feeding (Y/N)
If other feeding issues, describe them
Vascular malformations? (Y/N/unexplored)
If yes, revelation mode (screening, incidental finding, symptomatic etc)
If any other cardiovascular information, describe them
Precocious puberty ? (Y/N/too young)
New brain MRI since publication? (Y/N)
Brain MRI results (normal/abnormal: main findings)
Possibility of sending brain MRI images to us? (Y/N: CD, web link, etc)
Neurodeveloppement at last Follow-Up? (normal/slight, moderate, severe delay)
Mode of neurodevelopment evaluation (clinically, scales…)
Orthopedic disorders ? (Y/N)
If yes, detail orthopedic disorders (hip spasticity/scoliosis…)
Surgery done for orthopedic disorders or scheduled ? (Y/N)
If yes, detail which surgery
Autism spectrum disorder ? (Y/N)

**If yes, which critere/scale of diagnosis (ADOS, DSM….)**
**Behavioral disorders (Y/N)**
**Psychiatric therapies (Y/N)**
**If yes, detail them**
**Sleep disorders (Y/N)**
**Melatonin (Y/N)**
**Any other information ? Feel free to add it there**

B

**Paper reference (ex: Nabbout et al. 2024)**
**Patient identification in your paper (ex: patient 1) - *please use the same anonymisation that you used for the article***
**Age at last visit (year)**
**Active epilepsy at last follow-up (> 1 seizure/year) (Y/N)**
**If yes, add frequency**
**Seizure types at last follow-up (generalized/focal/both/unknown)**
**ASM at last follow-up? (Y/N)**
**If yes, please detail them (ex: sodium valproate, lamotrigine, Cannabidiol…)**
**Neurodeveloppement at last follow-up? (normal/slight, moderate, severe delay)**
**Mode of neurodevelopment evaluation (clinically, scales…)**
**Autism spectrum disorder ? (Y/N)**
**If yes, which critere/scale of diagnosis (ADOS, DSM….)**
**Other psychiatric disorder at last FU (Y/N)**
**If yes, precise type (depression/anxiety/behavior disorders…)**
**Psychiatric therapies (Y/N)**
**If yes, detail them**

Supplementary Table 2: Questionnaire sent to clinicians

A- For EIMFS and non-EIMFS DEE patients

B- For SHE and other patients

ADOS: Autism Diagnostic Observation Schedule, ASM: AntiSeizure Medications, DEE: Developmental and Epileptic Encephalopathy, DSM: Diagnostic and Statistical Manual of Mental Disorders, EIMFS: Epilepsy of Infancy with Migrating Focal Seizures, KD: Ketogenic Diet, MRI: Magnetic Resonance Imgaing, OFC: Occipital-Frontal Circumference, SD: Standard Deviation, SHE: Sleep-related Hypermotor Epilepsy, VNS = Vagus Nerve Stimulation

Patient's reference	Variant c. / p. / inheritance	Epilepsy syndrome	Sex	Age at pu-blication (y)	Sz onset (m)	Sz frequency at publication	Sz types (global) at publication	MRI at publication	Cognition at publication	Age at last visit (y)	Death since the publication (age (y) ; cause)	Active epilepsy at LFU	Seizure frequency at LFU	Seizure types at LFU	ASM at LFU ?	KD at LFU?	Epilepsy surgery since the publication ? (age (y) and type)	New brain MRI since publication ? (results)	Neurodeveloppement at LFU?
**Bonardi et al. 2021[1] (10)**	c.862G>A / p.Gly288Ser / De novo	EIMFS	M	1,4	6	Multiple daily	HemiC, TAS, eTCS, SE	WMA, frontal gliosis, thin CC and brainstem, fronto- temporo-insular CA, cerebellar atrophy, enlarged ventricles	Severe NDD	7	No	Yes	Yearly	GS, FS	Yes (LTG, VPA, PHB)	No	No	No	Severe to profound ID
**Bonardi et al. 2021(11)**	c.862G>A / p.Gly288Ser / NA	EIMFS	M	0,8	4	NA	HemiC, eTCS, SE	Normal (age NA)	Severe NDD	6	No	Yes	Yearly	GS, FS	Yes (VPA, STP, CLB)	No	No	Yes (3 yo: CA, subdural hygromas)	Severe to profound ID
**Bonardi et al. 2021(20°), Fitzgerald et al. 2019[2] (16)**	c.1429G>A / p.Ala477Thr / De novo	EIMFS	F	14	0,5	Daily	FS, Subclinical, eTCS, Spasms	Global CA, thin CC	Severe ID	18	Yes (18 ; respiratory infection)	Yes	Weekly	FM, spasms	Yes (Epidyolex, QUIN(weaning off-ultimately came off successfully without worsening seizures), VPA, CZP, BRV, CLB)	Yes, 4:1 ratio	No	No	Severe to profound ID
**Bonardi et al. 2021(31)**	c.2849G>A / p.Arg950Gln / De novo	EIMFS	M	18,5	5	Weekly	FS, TCS, SE	Bilateral temporal WMA	ID	24	No	Yes	Multiple daily	TS, rare TC	Yes (VPA, PHB, LTG, CBD (not commercial))	No	No	No	Severe to profound ID
**Bonardi et al. 2021(33°), Fitzgerald et al. 2019 (6)**	c.2881C>A / p.Arg961Ser / De novo	EIMFS	M	2	2	Yearly	HemiC, FIA, Subclinical, TCS, Myo	Normal (age NA)	Severe NDD	11	No	Yes	Yearly	FM (clonic)	Yes (LEV)	No	No	No	Severe to profound ID
**Cherian et al. 2021[3] (patient 5) IV-5**	c.2882G>A / p.Arg961His / inherited	EIMFS	F	7	3	Weekly	FM (3 m), FNMIA+Au (6 m), FM (4y), Gmyo, unknown onset TC Sz, MFS	5 yo: normal	Severe ID	11	No	Yes	Multiple daily	FM (tonic or clonic)	Yes (BRV, PHE, TPM)	Yes	No	No	Severe to profound ID
**Dilena et al. 2018[4] (patient 1)**	c.2849G>A / p.Arg950Gln / De novo	EIMFS	M	1	0,06	Multiple daily	Au	1st dol: normal	Severe NDD	7,5	No	Yes	Multiple daily	F migrating (tonic, versive, clonic, autonomic); FTB(TC)	Yes (LEV)	No	No	No	Severe to profound ID
**Dilena et al. 2018 (patient 2)**	c.2677G>A / p.Glu893Lys / De novo	EIMFS	M	2	0,03	Multiple daily	FM+Au, Spasms	NA	Severe NDD	6	Yes (6 ; severe pulmonary hemorrhage)	Yes	Multiple daily	FS, GS	Yes (QUIN, FBM, CBD, CLB, DZP)	No	No	No	Severe to profound ID
**Ferretti et al. 2022[5] (patient 1)**	c.337G>A / p.Val113Met / De novo	EIMFS	M	5,75	9	Daily	FM, Spasms	6 mo: thin CC	Severe NDD	7	Yes (7 ; respiratory infection)	Yes	Multiple daily	GS	Yes (TPM, LCS, QUIN, CLZ)	No	No	No	Severe to profound ID
**Ferretti et al. 2022 (patient 3)**	c.862G>A / p.Gly288Ser / De novo	EIMFS	M	2,5	1,3	NA	FM	2mo : normal	NDD	5	No	Yes	Multiple daily	GS	Yes (CBZ, PHE, QUIN)	No	No	No	Moderate NDD
**Ikeda et al. 2021[6] (patient 2), Ohba et al. 2015[7] (patient 2)**	c.808C>G / p.Gln270Glu / De novo	EIMFS	F	8	0,006	NA	FM	28 do: thin CC ; 7 mo: diffuse CA ; 35 mo: delayed myelination	Severe ID	14	No	Yes	Multiple daily	FS	Yes (PB, LEV, LTG, KBr)	No	No	No	Severe to profound ID

**Kawasaki et al. 2017[8] (patient 3), Ohba et al. 2015 (patient 7)**	c.1421G>A / p.Arg474Cys / De novo	EIMFS	M	8	0,5	Multiple daily	FM, Au	7 mo: normal	Severe ID	6,5	No	Yes	Multiple daily	FS, TS, automatic seizure	Yes (PHE)	No	No	No	Severe to profound ID
**Kohli et al. 2020[9]**	c.1420C>T / p. Arg474Cys / NA	EIMFS	F	0,83	NA	Multiple daily	FM	2 mo: delayed myelination, thin CC	Severe NDD	5	No	Yes	Multiple daily	FS, GS	Yes (PHE, VGV, LEV)	Yes	No	No	Severe NDD
**Kravetz et al. 2021[10]**	c.2795T>C / p.Phe932Ser / De novo	EIMFS	F	4	3	NA	FM	3 yo: subtle signs of CA, hypoplastic CC, WMA - more prominent in the left hemisphere.	NA	15	No	Yes	Monthly	FTB(TC)	Yes (TPM)	NA	NA	NA	Severe to profound ID
**Kuchenbuch et al. 2019[11] (patient 1), Barcia et al. 2012[12] (patient 2), Kim et al. 2014[13] (1)**	c.1283G>A / p.Arg428Gln / De novo	EIMFS	M	16,2	2	Weekly	FS, GS	5yo: Myelination delay, thin CC, CA	ID	23	No	Yes	Nighlty	GS (tonic)	No (Rivotril when needed)	No	No	No (CT scan: normal cerebral arteries; minimal bilateral symmetrical cerebellar atrophy; minimal frontal atrophy)	Severe to profound ID
**Kuchenbuch et al. 2019 (patient 10)**	c.1283G>A / p.Arg428Gln / De novo	EIMFS	F	7	NA	NA	NA	NA	Severe ID	12,4	Yes (13,4 ; respiratory infection)	Yes	Multiple daily	GS	No (Rivotril when needed)	Yes	No	No	Severe to profound ID
**Kuchenbuch et al. 2019 (patient 11)**	c.1420C>T / p.Arg474Cys / De novo	EIMFS	F	3,8	NA	NA	NA	NA	Severe NDD	6	No	Yes	Multiple daily	FM (tonic)	Yes (LTG, Rivotril when needed)	Yes	No	No	Severe to profound ID
**Kuchenbuch et al. 2019 (patient 14)**	c.1421G>A / p.Arg474His / De novo	EIMFS	M	2,1	0,03	NA	NA	NA	Severe NDD	9,4	No	Yes	Multiple daily	FM	Yes (CBD)	No	No	No	Severe to profound ID
**Kuchenbuch et al. 2019 (patient 16)**	c.2800G>A / p.Ala934Thr / De novo	EIMFS	M	0,9	NA	NA	NA	Early MRI: thinCC	Severe NDD	2,75	No	Yes	Multiple daily	FS	Yes (CBD)	No	No	No	Severe NDD
**Kuchenbuch et al. 2019 (patient 17), Merdariu et al. 2012[14]**	c.2688G>A / p.Met896Ile / De novo	EIMFS	F	8	2	Weekly	FIA, FC, auto	6 mo: mild enlargement of pericerebral spaces	Mild ID	15,25	No	Yes	Multiple weekly	Noctural, FS	Yes (LEV, Diacomit, Rivotril, Epidyolex)	No	No	No	ID
**Numis et al. 2018[15] (patient 2), Poisson et al. 2020[16] (patient 2), Cornet et al. 2021[17] (patient 15)**	c.776C>A / p.Ala259Asp / De novo	EIMFS	M	NA	0,03	Multiple daily	FM, Au	5 mo: severe diffuse thin CC, mild WMA ; MRS: normal	Profound ID	12	No	Yes	Multiple daily	Fmigrating, FIA, Auto	Yes (Cenobamate, Fycompa, RUF, GPT)	No	No	No	Severe to profound ID
**Ohba et al. 2015 (patient 3)**	c.1225C>T / p.Pro409Ser / De novo	EIMFS	F	5,3	2	NA	FS	2 mo: Mildly thin CC and CA ; 4 mo: subdural hematoma	Severe NDD	16	No	Yes	Multiple daily	NA	Yes (PHE, VPA, LEV, TPM, PER)	No	No	No	Severe to profound ID
**Ohba et al. 2015 (patient 4)**	c.862G>A / p.Gly288Ser / De novo	EIMFS	M	1,83	4	NA	FM	4mo: subdural hygroma ; 7 mo: diffuse CA	Severe NDD	1	Yes (1 ; respiratory infection)	Yes	Multiple daily	TS	Yes (LEV, KBr, PB, VPA)	Yes	Yes (1 ; VNS, no effect)	No	Severe NDD
**Ohba et al. 2015 (patient 5)**	c.1421G>A / p.Arg474His / De novo	EIMFS	M	2,25	1	NA	FM	2 mo: normal ; 1 yo: CA	Severe NDD	13	No	Yes	Multiple daily	TS	Yes (PB, LCM, BRV, RUF, KBr, PER, FFA)	No	No	Yes (8 yo: diffuse CA)	Severe to profound ID
**Ohba et al. 2015 (patient 6)**	c.2800G>A / p.Ala934Thr / De novo	EIMFS	M	6,16	0,13	NA	FM	2 mo: normal ; 2 yo: normal	Profound ID	17	No	Yes	Multiple weekly	NA	Yes (LEV, PHE, LTG)	No	No	No	Severe to profound ID
**Ohba et al. 2015 (patient 9)**	c.2771C>T / p.Pro924Leu / Inherited from	EIMFS	F	1,16	1,5	NA	InM	2 mo: normal	Severe NDD	12	No	Yes	Multiple yearly	FS	Yes (NZP, acetazolamide, NaBr)	No	No	No	Severe to profound ID

	the mother who carried a somatic mosaic mutation																		
**Trivisano et al. 2025[18] (patient 1)**	c.1193G>A / p.Arg398Gln / De novo	EIMFS	M	2,5	NA	Multiple daily	InM	NA	Severe NDD	3	No	Yes	Multiple daily	GS	Yes (TPM, CBD, CLB)	No	No	No	Moderate NDD
**Trivisano et al. 2025 (patient 3)**	c.1066C>T / p.Arg356Trp / De novo	EIMFS	F	15	NA	Multiple daily	NA	NA	Severe ID	15	No	Yes	Multiple daily	GS	Yes (PHE, LRZ, CLB, RUF, LCS)	No	No	No	Severe to profound ID
**Yoshitomi et al. 2019[19] (patient 1)**	c.1283G>A / p.Arg428Gln / De novo	EIMFS	M	1,7	1	Multiple daily	FM	NA	Severe NDD	9	No	Yes	Multiple daily	FS (tonic)	Yes (LCM, KBr, QUIN)	Yes	No	Yes (Abnormal. Non-specific diffuse CA, moderate. Delayed myelination.)	Severe to profound ID
**Yoshitomi et al. 2019 (patient 2)**	c.2800G>A / p.Ala934Thr / De novo	EIMFS	F	NA	2	Multiple daily	FM	NA	Severe NDD	10	No	Yes	Multiple daily	FS (tonic)	No (QUIN)	No	No	Yes (Abnormal. Non-specific diffuse CA, moderate. Delayed myelination.)	Severe to profound ID
**Yoshitomi et al. 2019 (patient 3)**	c.862G>A / p.Gly288Ser / De novo	EIMFS	M	NA	2	Multiple daily	FM, Au	NA	Severe NDD	4	No	Yes	Multiple daily	FS (tonic)	No (QUIN)	No	Yes (3,75 ; total callosotomy)	Yes (Abnormal. Non-specific diffuse CA,mild. Delayed myelination.)	Severe to profound ID
**Bonardi et al. 2021(40)**	c.2686A>G / p.Met896Val / De novo	DEE	F	24	6	Yearly	Spasms, Myo	7 mo: CA	Severe ID	31	No	Yes	Variable: monthly to daily	FS, GS	Yes (VPA, LTG, CNZ, LEV)	NA	No	No	Severe to profound ID
**Bonardi et al. 2021(48)**	c.940A>G / p.Thr314Ala / De novo	DEE	F	31,5	15	Multiple daily	HMS, FIA, eTCS, TCS, TS, A-abs, SE, FLS	7 yo: normal ; 19 yo: normal ; 6y: PET: normal	Severe ID	37	No	Yes	Monthly	FIA, noctural HMS, GTC	Yes (VPA, PHE, STP, Thylloflazepate)	No	No (in 2018 VNS implantation)	No	Severe to profound ID
**Chong et al. 2016[20], Ohba et al. 2015 (patient 11)**	c.1283G>A / p.Arg428Gln / De novo	DEE	M	6	1,5	Multiple daily	NA	1 mo: normal ; 2,5 yo: hypoplastic CC, delayed myelination, left lateral ventricular dilatation	Profound ID	15	No	Yes	Multiple daily	FS, GS	Yes (LEV, LTG, VPA)	No	No (VNS at age 2)	No	Severe to profound ID
**Datta et al. 2019[21] (patient 2)**	c.1420C>G / p.Arg474Gly / De novo	DEE	M	NA	0,75	Multiple daily	FM	2,5 yo: normal	NDD	9,5	No	Yes	Multiple weekly	FM, FIA	Yes (VPA, PHB, LAC, LEV)	No	No	Yes (Asymmetric prominece of sulcal spaces L hemisphere, susupicious for left frontal, temporal and Parieto occipital cortical dysplasia.)	Moderate ID
**Filareto et al. 2025[22] (case 6)**	c.785G>A / p.Arg262Gln / De novo	DEE	M	NA	36	NA	NA	NA	Severe NDD	8	No	Yes	Multiple daily	FS, myoclonic seizure and focal with fall	Yes (oxCBZ, VPA, Cenobamate, CNZ)	NA	NA	NA	Severe to profound ID
**Fukuoka et al. 2017[23]**	c.1955G>T / p.Gly652Val / De novo	DEE	M	2,6	5	Multiple daily	Spasms	5 mo: normal	NDD	12	No	Yes	Monthly	GS	Yes (QUIN, VPA)	No	Yes (4 ; total callosotomy)	Yes (Disconnected CC)	Severe to profound ID
**Ikeda et al. 2021[6] (patient 1), Ohba et al. 2015 (patient 1)**	c.1420C>T / p.Arg474Cys / De novo	DEE	M	12	0	NA	FS	2 mo: thin CC ; 10 mo: delayed myelination and CA	Severe ID	15	Yes (15 ; severe pulmonary hemorrhage)	Yes	Multiple daily	FM (tonic)	Yes (PB, LCM, KBr)	No	No	No	Severe to profound ID

**Krygier et al. 2024[24] (72 F)**	c.1193G>A / p.Arg398Gln / NA	DEE	F	5	6	NA	FM, FIA	Normal (age NA)	NDD	7	No	No			Yes (PRIMIDONUM)	Yes	No	No	Moderate ID
**Krygier et al. 2024 (87M)**	c.862G>A / p.Gly288Ser / NA	DEE	M	5	1	NA	FM	Normal	NDD	7	No	Yes	Weekly	FS, GS	Yes (PRIMIDONUM)	Yes	No	No	Severe to profound ID
**Shchubelka et al. 2024[25] (16)**	c.1309C>T / p.Leu437Phe / NA	DEE	NA	NA	NA	NA	NA	NA	NA	3	No	Yes	Multiple monthly	FS, GS	Yes (VPA)	No	No	No	Moderate NDD
**Shchubelka et al. 2024 (17)**	c.784C>T / p.Arg262Trp / NA	DEE	NA	NA	NA	NA	NA	NA	NA	10	No	Yes	Multiple yearly	GS	Yes (VPA, LTG, CBD)	No	No	No	Severe to profound ID
**Tsang et al. 2018[26] (patient 7)**	c.1038C>A (VUS) / p.Phe346Leu / inherited (paternal mosaicism)	DEE	M	0,75	3	NA	NA	< 9 mo: CA and delayed myelination	NDD	9	No	Yes	Monthly	FS, GS	Yes (CBZ, LEV)	No	No	No	Severe to profound ID
**Vanderver et al. 2014[27]**	c.2794T>A / p.Phe932Ile / De novo (variant LoF et pas GoF)	DEE	M	10	1	NA	Gmyo	1 mo: severe delayed myelination ; 7 yo: progression of myelination but at levels that are still severely reduced.	Severe ID	19	No	Yes	Weekly	FS	Yes (TPM, LTG)	No	No	No	Severe to profound ID
**Yamamoto et al. 2023[28] - patient 2**	c.1421G>A / p.Arg474His / De novo	DEE	F	4	1	Daily	FM, Spasms	NA	Severe NDD	5,9	Yes (7,9 ; uncertain (sudden death))	Yes	Multiple daily	FS, GS	Yes (LTG, clorazepate)	No	No	No	Severe NDD
**Yoshitomi et al. 2019[19] (patient 4)**	c.1420C>T / p.Arg474Cys / de novo	DEE	M	NA	1	Multiple daily	FM	NA	Severe NDD	15	Yes (15 ; severe pulmonary hemorrhage)	Yes	Multiple daily	FS (tonic)	Yes (PB, LCM, KBr)	No	No	Yes (Abnormal. Non-specific diffuse CA,mild. Delayed myelination.)	Severe to profound ID
**Derry et al. 2008[29] (B II-5), Heron et al. 2012[30] (A), Mullen et al. 2017[31] (2)**	c.2782C>T / p.Arg928Cys / inherited	SHE	M	54	180	Multiple nighlty	FLS, auto, GTC	Normal (age NA)	Mild ID	60	NA	Yes	Multiple yearly	Sleep-related and awake hypermotor seizures with choking, gasping for breath, dystonia, oral automatisms	Yes (VPA, TPM, CNZ)	NA	NA	NA	Borderline ID
**Derry et al. 2008 (B III-2), Heron et al. 2012 (A), Mullen et al. 2017 (5)**	c.2782C>T / p.Arg928Cys / inherited	SHE	F	21	36	Multiple nighlty	FLS, auto	Normal (age NA)	Mild ID	39	NA	Yes	1/2-3 night	Sleep-related hypermotor seizures; GTC	Yes (CNZ, CBZ, TPM, VPA, PHE)	NA	NA	NA	Moderate ID
**Derry et al. 2008 (B III-6), Heron et al. 2012 (A), Mullen et al. 2017 (3)**	c.2782C>T / p.Arg928Cys / inherited	SHE	F	6	33	Multiple nighlty	FLS, auto	33 mo: normal	Mild to moderate ID	26	NA	Yes	Multiple weekly	Hypermotor seizures; tonic seizures; GTC	Yes (CNZ, CBZ, TPM, VPA, PHE)	NA	NA	NA	Severe to profound ID
**Derry et al. 2008 (B III-7), Heron et al. 2012 (A), Mullen et al. 2017 (4)**	c.2782C>T / p.Arg928Cys / inherited	SHE	M	5	12	Multiple nighlty	FLS, auto	NA	Normal	25	NA	Yes	Weekly	Sleep-related and awake hypermotor seizures; FTB(TC)	Yes (TPM, CBZ, CNZ, LEV)	NA	NA	NA	Mild ID
**Derry et al. 2008 (family B II-4), Heron et al. 2012 (family A),**	c.2782C>T / p.Arg928Cys / inherited	SHE	M	43	24	Multiple nighlty	FLS, auto	Normal (age NA)	Mild ID	49	NA	Yes	Nightly	Sleep-related hypermotor seizures	Yes (VPA, TPM, CBZ)	NA	NA	NA	Mild ID

**Mullen et al. 2017 (1)**																			
**Héron et al. 2012 (case D)**	c.2688G>A / p.Met896Ile / De novo	SHE	M	NA	108	NA	NA	NA	Normal	49	NA	Yes	Monthly	Sleep-related and awake focal impaired awareness seizures; tonic-clonic seizures reported once	Yes (VPA, LTG, TPM, CNZ)	NA	NA	NA	Borderline ID (NPA)
**Hildebrand et al. 2016[32] (9092), Mullen et al. 2017 (6)**	c.2849G>A / p.Arg950Gln / De novo	SHE	M	28	36	NA	Focal dyscognitive (13y), Aura (3y)	Normal (age NA)	Mild ID	38	NA	Yes	Multiple daily	Hypermotor seizures with and without impaired awareness; FTB(TC); focal motor status epilepticus	Yes (CBZ, CNZ, Midazolam)	NA	NA	NA	Mild ID
**Licchetta et al. 2020[33]**	c.2800G>A / p.Ala934Thr / De novo	SHE	F	NA	108	NA	NA	NA	ID	29	NA	No			Yes (CBZ, LCS)	NA	NA	NA	Mild ID
**Cataldi et al. 2019[34] (cas index)**	c.2896G>A / p.Ala966Thr / inherited	SHE + EIMFS	M	NA	24	NA	FLS (noctural), migrating focal (diurnal)	24 mo: multiple very small cerebral cavernomas with no sign of previous bleedings.	Mild ID	NA	NA	Yes	NA	NA	NA	NA	NA	NA	Mild ID
**Heron et al. 2012 (patient III2-familyB)**	c.2386T>C / p.Tyr796His / inherited	SHE/ FCD Ib	M	35	36	NA	FLS, nocturnal wandering	Focal cortical dysplasia Ib	Learning difficulties	31	NA	Yes	Yearly	seizures during wakefulness with staring	Yes (CBZ, VPA, PHE, DZP)	NA	NA	NA	ID
**Cherian et al. 2021 (patien t 1) II-5**	c.2882G>A / p.Arg961His / NA	Focal epilepsy	F	64	168	NA	TLS (staring, feeling of terror, stightness).	NA	Normal	64	NA	Yes	Monthly	FNM, GS once	No	NA	NA	NA	Normal
**Cherian et al. 2021 (patien t 2) III-11**	c.2882G>A / p.Arg961His / inherited	Focal epilepsy	F	40	120	Multiple daily (FNM + Au) - Multiple yearly (FGTC)	FNM + Au (presumed insular/opercular onset), FGTC	19 yo: normal ; 24 yo: normal ; 37 yo: normal	Normal	41	NA	Yes	Monthly	FNM; FBTCS; Questionable auditory aura (from wakefulness)	Yes (CBZ)	NA	NA	NA	Normal
**Cherian et al. 2021 (patien t 4) III-14**	c.2882G>A / p.Arg961His / inherited	Focal epilepsy	F	30	216	Multiple nighlty	FNM + Au (presumed insular/opercular onset)	24 yo: normal	Cognitive variability	31	NA	Yes	Monthly	FNM; FBTCS	Yes (PHE, BRV, TPM)	NA	NA	NA	Normal
**Mosca et al. 2024[35], Filareto et al. 2025[22] (case 5)**	c.2809A>G / p.Ser937Gly / De novo	Focal epilepsy	F	20	36	None	TLS (staring, feeling of terror, swallowing, abdomen tightness).	3 yo: normal ; 18 yo: normal	Normal	21	fNo	Yes	Yearly	FS	Yes (oxCBZ, CLZ)	NA	NA	No	Mild ID

Supplementary Table 3: Individual neurological features of patients with clinical updates.

Abbreviations :A-Abs: Atypical Absences, ASM: AntiSeizure Medications, Au: Autonomic, BRV: Brivacetam, CA: Cerebral Atrophy, CBD: Cannabidiol, CBZ: Carbamazepine, CC: Corpus Callosum, CLB: Clobazam, CLZ: Clorazepam, CNZ: Clonazepam, CT-Scan: Computed Tomography scan, DEE: Developmental Epileptic Encephalopathy, DOI: day of life, DZP: Diazepam, EIMFS: Epilepsy of Infancy with Migrating Focal Seizure, eTCS: evolving to Tonic Clonic Seizure, FBM: Felbamate, FIA: Focal Impaired Awarenesse, FLS: frontal lobe seizures, F: Focal, FTB(TC): Focal To Bilateral (Tonic-Clonic), FNM: Focal Non Motor, FNMIA: Focal Non Motor Impaired Awareness, FM: Focal Motor, FS: Focal Seizures, FU: Follow-Up, GMyo: Generalized Myoclonia, GS: Generalized

Seizures, GPT: Gabapentine,HemiC: hemiclonic, ID: Intellectual Disability, KBr: Potassium Bromide, KD: Ketogenic Diet, LAC: Lacosamide, LEV: Levetiracetam, LFU: Last Follow-Up, LCM: Lacosamide, LRZ: Lorazepate, LTG: Lamotrigine, MFS: Migrating Focal Seizure, M/F: Male/Female, mo: month old, MRI: Magnetic Resonance Imaging, MRS: Magnetic Resonance Spectroscopy, Myo: myoclonus, NA: Non Available, NaBr: Sodium Bromide; NDD: Neurodevlopmental Delay, NZP: Nitrazepam, oxCBZ: Oxcarbazepinde, PER: Perampanel, PHB: Phenobarbital,PHE: Phenytoine, QUIN: quinidine, RUF: Rufinamide, SE: Statuts Epilepticus, SHE: Sleep-related Hypermotor Epilepsy, STP: Stiripentol, Sz: Seizure, TCS: Tonic Clonic Seizure, TLS: Temporal Lobe Seizure, TPM: Topiramate, TS: Tonic Seizures, VGV: Vigabatrin, VNS: Vagus Nerve Stimulation, VPA: Valproate, WMA: White Matter Abnormalities, yo: year old

Patient's reference	Variant c. / p. / inheritance	Epilepsy syndrome	Sex	Age at last visit (y)	Death since the publication (age (y) ; cause)	Feeding disorders	Vascular malformations (detail / revelation mode)	Precocious puberty ?	Orthopedic features	Behavioral disorders	Autism spectrum disorder (diagnosis scale)	Other psychiatric disorder associated	Psychiatric therapies	Sleep disorders
**Bonardi et al. 2021(10)**	c.862G>A / p.Gly288Ser / De novo	EIMFS	M	7	No	Yes (GT)	NA	No	No	No	No	NA	No	No
**Bonardi et al. 2021(11)**	c.862G>A / p.Gly288Ser / NA	EIMFS	M	6	No	Yes (GT)	NA	No	Yes (Hip spasticity)	No	No	NA	No	No
**Bonardi et al. 2021(20°), Fitzgerald et al. 2019 (16)**	c.1429G>A / p.Ala477Thr / De novo	EIMFS	F	18	Yes (18 ; respiratory infection)	Yes (GT)	NA	No	Yes (Spastic quad, scoliosis, osteopenia, neuromuscular hip dysplasia / Right hip femoral head and neck resection with valgus osteotomy, spinal fusion)	No	No (too impaired to assess)	NA	NA	No
**Bonardi et al. 2021(31)**	c.2849G>A / p.Arg950Gln / De novo	EIMFS	M	24	No	Yes (eats blended food)	No	No	No	Yes	No	NA	No	No
**Bonardi et al. 2021(33°), Fitzgerald et al. 2019 (6)**	c.2881C>A / p.Arg961Ser / De novo	EIMFS	M	11	No	No	NA	No	Yes (Spastic quad, neuromuscular hip dysplasia / Bilateral hip surgery)	No	No (too impaired to assess)	NA		No
**Cherian et al. 2021 (patient 5) IV-5**	c.2882G>A / p.Arg961His / inherited	EIMFS	F	11	No	Yes (GT)	No	Yes	Yes (Scoliosis, Superolaterally subluxed right femoral head that reduces in frog-leg view, generalized osteopenia)	No	No (too delayed to say)	NA	No	Yes (insomnia but likely due to frequent seizures)
**Dilena et al. 2018 (patient 1)**	c.2849G>A / p.Arg950Gln / De novo	EIMFS	M	7,5	No	Yes (GT)	No	No	No	No	No	NA		Yes (Melatonin)
**Dilena et al. 2018 (patient 2)**	c.2677G>A / p.Glu893Lys / De novo	EIMFS	M	6	Yes (6 ; severe pulmonary hemorrhage)	Yes (GT, GERD, dysphagia)	Yes (Ectasia of the bronchial arteries and the main pulmonary artery / Symptomatic (recurrent respiratory infections))	Too young	Yes (Bilateral cervico-diaphyseal valgus with incomplete coverage of the femoral head (>right))	No	No	NA	No	Yes (Melatonin)

**Ferretti et al. 2022 (patient 1)**	c.337G>A / p.Val113Met / De novo	EIMFS	M	7	Yes (7 ; respiratory infection)	Yes (GT)	No	No	Yes (Mild kyphoscoliosis, bilateral valgus-pronated foot)	No	No	NA	No	No
**Ferretti et al. 2022 (patient 3)**	c.862G>A / p.Gly288Ser / De novo	EIMFS	M	5	No	Yes (delay in feeding and swallowing skills)	No	Too young	No	No	No	NA	No	No
**Ikeda et al. 2021 (patient 2), Ohba et al. 2015 (patient 2)**	c.808C>G / p.Gln270Glu / De novo	EIMFS	F	14	No	Yes (GT)	Yes (SPCA / Symptomatic (pulmonary hemorrhage))	No	Yes (Hip dislocation, limb joint contracture)	No	No	NA	No	No
**Kawasaki et al. 2017 (patient 3), Ohba et al. 2015 (patient 7)**	c.1421G>A / p.Arg474Cys / De novo	EIMFS	M	6,5	No	Yes (GT)	Yes (Systemic to pulmonary collateral arteries / Incidental finding)	No	No	NA	No	NA		Yes
**Kohli et al. 2020**	c.1420C>T / p. Arg474Cys / NA	EIMFS	F	5	No	Yes (GT, GERD, dysphagia, feeding intolerance)	Yes (SPCA)	Too young	Yes (Scoliosis, hips spasticity, feet inversion)	No	No	NA	No	Yes
**Kravetz et al. 2021**	c.2795T>C / p.Phe932Ser / De novo	EIMFS	F	15	No	NA	NA	NA	NA	No	No	NA	No	NA
**Kuchenbuch et al. 2019 (patient 1), Barcia et al. 2012 (patient 2), Kim et al. 2014 (1) - CONSOLIDATION**	c.1283G>A / p.Arg428Gln / De novo	EIMFS	M	23	No	Yes (eats blended food)	NA	No	Yes (Hip dislocation (2), Scoliosis / Sinal arthrodesis (scoliosis))	No	NA	NA	No	NA
**Kuchenbuch et al. 2019 (patient 10) - CONSOLIDATION**	c.1283G>A / p.Arg428Gln / De novo	EIMFS	F	12,4	Yes (13,4 ; respiratory infection)	Yes (GT, constipation)	NA	No	Yes (Hip dislocation, Severe scoliosis / Spinal arthrodesis, hip dislocation (2))	No	NA	NA	No	Yes (Melatonin)
**Kuchenbuch et al. 2019 (patient 11) - STORMY**	c.1420C>T / p.Arg474Cys / De novo	EIMFS	F	6	No	Yes (GT)	NA	Too young	Yes (Hip dislocation (1), Scoliosis)	No	NA	NA	No	No
**Kuchenbuch et al. 2019 (patient 14) - STORMY**	c.1421G>A / p.Arg474His / De novo	EIMFS	M	9,4	No	Yes (constipation)	NA	Too young	Yes (Hip dislocation (1), Scoliosis)	No	NA	NA	No	No
**Kuchenbuch et al. 2019 (patient 16) - STORMY**	c.2800G>A / p.Ala934Thr / De novo	EIMFS	M	2,75	No	Yes (painful dyspepsia)	NA	Too young	Yes (Hip dislocation without pain (1))	No	No	NA	No	No
**Kuchenbuch et al. 2019 (patient 17), Merdariu et al. 2012 - CONSOLIDATION after Sz free period**	c.2688G>A / p.Met896Ile / De novo	EIMFS	F	15,25	No	NA	NA	No	NA	No	NA	NA	No	No
**Numis et al. 2018 (patient 2), Poisson et al. 2020 (patient**	c.776C>A / p.Ala259Asp / De novo	EIMFS	M	12	No	Yes (GT, GERD, constipation)	No	No	Yes (Hip dysplasia (2), scoliosis, spasticity with contractures and clonus, mild bilateral	Yes	No (too impaired to assess)	Agitation started 10/2023, managed with	Yes (Gabapentin, clonidine)	Yes (no regular cycling)

**2), Cornet et al. 2021 (patient 15)**									equinus / Hip abductor surgery 4/2018)			gabapentin and clonidine		
**Ohba et al. 2015 (patient 3)**	c.1225C>T / p.Pro409Ser / De novo	EIMFS	F	16	No	Yes (GT)	NA	No	Yes (Right femoral fracture, Scoliosis)	No	No	NA	No	Yes
**Ohba et al. 2015 (patient 4)**	c.862G>A / p.Gly288Ser / De novo	EIMFS	M	1	Yes (1 ; respiratory infection)	Yes (GT)	No	Too young	No	No	No	NA	No	No
**Ohba et al. 2015 (patient 5)**	c.1421G>A / p.Arg474His / De novo	EIMFS	M	13	No	Yes (GT)	Yes (Aorto-pulmonary collateral artery / Symptomatic)	No	No	No	No	NA		Yes (Melatonin)
**Ohba et al. 2015 (patient 6)**	c.2800G>A / p.Ala934Thr / De novo	EIMFS	M	17	No	Yes (GT)	NA	No	Yes (Hip dislocation (1), Right supracondylar femoral fracture, Scoliosis)	No	No	NA	No	No
**Ohba et al. 2015 (patient 9)**	c.2771C>T / p.Pro924Leu / Inherited from the mother who carried a somatic mosaic mutation	EIMFS	F	12	No	Yes (eats blended food)	NA	No	Yes (Hip dislocation (1), scoliosis / Left hip arthroplast was performed at age 9)	No	No	NA	No	Yes (Melatonin)
**Trivisano et al. 2025 (patient 1)**	c.1193G>A / p.Arg398Gln / De novo	EIMFS	M	3	No	Yes (GT)	No	Too young	No	No	No	NA	No	Yes (Melatonin)
**Trivisano et al. 2025 (patient 3)**	c.1066C>T / p.Arg356Trp / De novo	EIMFS	F	15	No	Yes (GT)	Yes (Angiodysplasia of the bronchial arteries and anomalous pulmonary venous return, abdominal vascular anomalies (celiac tripod ectasia, superior mesenteric arteryectasia, splenic arteryectasia), treated with embolizations / Symptomatic (hemoptysis/hemoptysis episodes))	No	Yes (Right hip dislocation, feet in talipes valgus, knees in valgus, scoliosis / Tenotomy of the adductors and psoas bilaterally; stretching of the medial flexors bilaterally	No	No	NA	No	Yes (lorazepam)
**Yoshitomi et al. 2019 (patient 1)**	c.1283G>A / p.Arg428Gln / De novo	EIMFS	M	9	No	Yes (GT, vomiting)	No	No	Yes (Contracture of bil ankle joints. Mild scoliosis. Acetabular dysplasia.)	No	No (too serverly delayed to diagnose ASD)	NA	No	No
**Yoshitomi et al. 2019 (patient 2)**	c.2800G>A / p.Ala934Thr / De novo	EIMFS	F	10	No	Yes (GT, vomiting, GERD)	No	No	No	No	No (too serverly delayed to diagnose ASD)	NA	No	No
**Yoshitomi et al. 2019 (patient 3)**	c.862G>A / p.Gly288Ser / De novo	EIMFS	M	4	No	Yes (NA)	NA	No	No	No	No (too serverly delayed to diagnose ASD)	NA	No	No
**Bonardi et al. 2021(40)**	c.2686A>G / p.Met896Val / De novo	DEE	F	31	No	Yes (eats blended food)	NA	No	Yes (Feet deformity / hip spasticity)	Yes	NA	NA	Yes (Risperidone)	Yes

**Bonardi et al. 2021(48)**	c.940A>G / p.Thr314Ala / De novo	DEE	F	37	No	Yes (mild dysphagia, eats blended food)	No	No	Yes (Scoliosis)	No	Not diagnosed	NA		No
**Chong et al. 2016, Ohba et al. 2015 (patient 11)**	c.1283G>A / p.Arg428Gln / De novo	DEE	M	15	No	Yes (GT)	NA	No	Yes (Hip dislocation, Scoliosis)	No	No	NA	No	No
**Datta et al. 2019 (patient 2)**	c.1420C>G / p.Arg474Gly / De novo	DEE	M	9,5	No	No	No	No	Yes (Right foot everted when walking)	NA	NA	NA	NA	No
**Filareto et al. 2025 (case 6)**	c.785G>A / p.Arg262Gln / De novo	DEE	M	8	No	NA	NA	NA	NA	Yes	No	Severe behaviour disorder characterized by hyperactivity, irritability and several stereotypes	Yes (Xenazina at low dosage for oral dyskinesias)	NA
**Fukuoka et al. 2017**	c.1955G>T / p.Gly652Val / De novo	DEE	M	12	No	Yes (GT)	No	Too young	Yes (Scoliosis)	No	No	NA	No	Yes
**Ikeda et al. 2021 (patient 1), Ohba et al. 2015 (patient 1)**	c.1420C>T / p.Arg474Cys / De novo	DEE	M	15	Yes (15 ; severe pulmonary hemorrhage)	Yes (GT)	Yes (SPCA / Symptomatic (pulmonary hemorrhage))	No	Yes (Scoliosis)	No	No	NA	No	No
**Krygier et al. 2024 (72 F)**	c.1193G>A / p.Arg398Gln / NA	DEE	F	7	No	No	No	No	No	Yes	No	NA	No	No
**Krygier et al. 2024 (87M)**	c.862G>A / p.Gly288Ser / NA	DEE	M	7	No	No	No	No	No	Yes	No	NA	No	No
**Shchubelka et al. 2024 (16)**	c.1309C>T / p.Leu437Phe / NA	DEE	NA	3	No	Yes (GT, food selectivity)	NA	Too young	Yes (Scoliosis, connective tissue disorder clinically)	Yes	Yes (ADOS)	NA	Yes (Risperidone)	Yes (Melatonin)
**Shchubelka et al. 2024 (17)**	c.784C>T / p.Arg262Trp / NA	DEE	NA	10	No	NA	NA	Too young	NA	Intellectual disability	No	NA	No	No
**Tsang et al. 2018 (patient 7)**	c.1038C>A (VUS) / p.Phe346Leu / inherited (paternal mosaicism)	DEE	M	9	No	Yes (GT, dysphagia)	No	No	Yes (Hip dislocation (2), Scoliosis)	No	No	NA	No	No
**Vanderver et al. 2014**	c.2794T>A / p.Phe932Ile / De novo (variant LoF et pas GoF)	DEE	M	19	No	Yes (GT, constipati on)	No	No	Yes (Neuromuscular scoliosis (right thoracolumbar), bilateral knee clunking or crepitus, right ACL abnormality, osteopenia)	No	No	NA	No	Yes (Melatonin)
**Yamamoto et al. 2023 - patient 2**	c.1421G>A / p.Arg474His / De novo	DEE	F	5,9	Yes (7,9 ; uncertain (sudden death))	Yes (eats blended food)	Yes (SPCA / Screening)	No	No	No	No	NA	No	No
**Yoshitomi et al. 2019 (patient 4)**	c.1420C>T / p.Arg474Cys / de novo	DEE	M	15	Yes (15 ; severe pulmonary hemorrhage)	Yes (GT)	Yes (SPCA / Symptomatic (nasal bleeding, hemoptysis))	No	Yes (Acetabular dysplasia. Severe scoliosis)	No	No (too serverly delayed to diagnose ASD)	NA	No	No

**Derry et al. 2008 (B II-5), Heron et al. 2012 (A), Mullen et al. 2017 (2)**	c.2782C>T / p.Arg928Cys / inherited	SHE	M	60	NA	NA	NA	NA	NA	NA	No	Mood disorder	Yes (Doxepin)	NA
**Derry et al. 2008 (B III-2), Heron et al. 2012 (A), Mullen et al. 2017 (5)**	c.2782C>T / p.Arg928Cys / inherited	SHE	F	39	NA	NA	NA	NA	NA	Yes	No	Rapid cyclic unipolar depression	Yes (Duloxetine)	NA
**Derry et al. 2008 (B III-6), Heron et al. 2012 (A), Mullen et al. 2017 (3)**	c.2782C>T / p.Arg928Cys / inherited	SHE	F	26	NA	NA	NA	NA	NA	NA	No	None	No	NA
**Derry et al. 2008 (B III-7), Heron et al. 2012 (A), Mullen et al. 2017 (4)**	c.2782C>T / p.Arg928Cys / inherited	SHE	M	25	NA	NA	NA	NA	NA	No	No	None	No	NA
**Derry et al. 2008 (family B II-4), Heron et al. 2012 (family A), Mullen et al. 2017 (1)**	c.2782C>T / p.Arg928Cys / inherited	SHE	M	49	NA	NA	NA	NA	NA	NA	No	Anxiety	No	NA
**Héron et al. 2012 (case D)**	c.2688G>A / p.Met896Ile / De novo	SHE	M	49	Yes (49 : glioblastoma)	NA	NA	NA	NA	NA	No	NA	Yes (Paroxetine, olanzapine, valium)	NA
**Hildebran d et al. 2016 (9092), Mullen et al. 2017 (6)**	c.2849G>A / p.Arg950Gln / De novo	SHE	M	38	NA	NA	NA	NA	NA	NA	Yes (Level 2 ASD using the ADOS at an autism clinic (30 y))	Recurrent psychosis (last at 38 y), anxiety, obsessive compulsive disorder	Yes (Quetiapine, diazepam)	NA
**Licchetta et al. 2020**	c.2800G>A / p.Ala934Thr / De novo	SHE	F	29	NA	NA	NA	NA	NA	NA	No	None	No	No
**Cataldi et al. 2019 (cas index)**	c.2896G>A / p.Ala966Thr / inherited	SHE + EIMFS	M	NA	NA	NA	NA	NA	NA	NA	No	None	No	NA
**Heron et al. 2012 (patient III2-familyB)**	c.2386T>C / p.Tyr796His / inherited	SHE/ FCD Ib	M	31	NA	NA	NA	NA	NA	NA	No	Schizophrenia, depression	Yes (Quetiapine, Clotiapine, Carbolithium, Diazepam)	NA
**Cherian et al. 2021 (patien t 1) II-5**	c.2882G>A / p.Arg961His / NA	Focal epilepsy	F	64	NA	NA	NA	NA	NA	NA	No	None	No	NA
**Cherian et al. 2021 (patien t 2) III-11**	c.2882G>A / p.Arg961His / inherited	Focal epilepsy	F	41	NA	NA	NA	NA	NA	NA	No	None	No	NA
**Cherian et al. 2021 (patien t 4) III-14**	c.2882G>A / p.Arg961His / inherited	Focal epilepsy	F	31	NA	NA	NA	NA	NA	NA	No	None	No	NA
**Mosca et al. 2024, Filareto et al. 2025 (case 5)**	c.2809A>G / p.Ser937Gly / De novo	Focal epilepsy	F	21	No	NA	NA	NA	NA	Yes	No	Depression and anxiety, improved after	Yes (Fluoxetine)	NA

	treatment with Fluoxetine

Supplementary Table 4 : Individual extra-neurological features of patients with clinical updates.

ACL: Anterior Cruciate Ligament, ADOS: Autism Diagnostic Observation Schedule, ASD: Autism Spectrum Disorder, DEE: Developmental and Epileptic Encephalopathy, EIMFS: Epilepsy of Infancy with Migrating Focal Seizures, FCD: Focal Cortical Dysplasia, GERD: Gastro-oesophageal reflux, GT: Gastrostomy, M/F: Male/Female, NA: Non Available, SHE: Sleep-related Hypermotor Epilepsy, SPCA: Systemic to pulmonary collateral arteries, y: yes

		EIMFS	DEE	SHE	Others	
Nter	p.Arg85Ser		1			
	p.Val113Met	1				
	p.Arg133His				1	
	p.Gly243Ser	1				
S5	p.His257Asp	1				GOF (Hinckley 2023[36])
S5	p.Ala259Asp	1				GOF (Numis 2018[15])
S5	p.Arg262Gln	1	1			GOF (Hinckley 2023)
S5	p.Arg262Trp		1			GOF (Hinckley 2023)
**S5**	**p.Met267Thr**	**3**				
**S5**	**p.Gln270Glu**	**3**				GOF (Hinckley 2023)
S5	p.Val271Phe	2				GOF (Hinckley 2023, McTague 2018[37])
pore	p.Leu274Ile	2				GOF (Hinckley 2023, McTague 2018)
**pore**	**p.Gly288Ser**	**24**	**7**	**2**	**2**	GOF (Hinckley, Rizzo)
S6	p.Thr314Ala		1			
S6	p.Ala338Gly	1				
S6	p.Val340Met				1	LOF (Hinkley)
	p.Phe346Leu	2	1			GOF (Hinckley 2023, McTague 2018)
**RCK1**	**p.Arg356Trp**	**4**				GOF (Trivisano 2025[18])
RCK1	p.Cys377Ser	1	1			
**RCK1**	**p.Arg398Gln**	**5 (6*)**	**5**	**6 (13*)**	**1**	GOF (Hinckley 2023, Milligan 2014[38], Trivisano 2025)
RCK1	p.Arg398Leu	1				
RCK1	p.Pro409Ser	1	1			GOF (Hinkley 2023, Barcia 2012[12])
**RCK1**	**p.Arg428Gln**	**21**	**1**			GOF (Hinkley 2023, Mikati 2015[39], Milligan 2014)
RCK1	p.Ser435Cys	1				
RCK1	p.Leu437Phe	1	2			
RCK1	p.Asn449Ser			1		
**RCK1**	**p.Arg474Cys**	**10**	**5**			LOF (Hinckley)
RCK1	p.Arg474Gly		1			

**RCK1**	**p.Arg474His**	**18**	**7**	**1**		GOF (Hinckley 2023, Trivisano 2025)
RCK1	p.Arg474Lys	1				
RCK1	p.Trp476Arg	2				
RCK1	p.Ala477Thr	2				GOF (Hinckley 2023)
RCK1	p.Asp480Asn	2				
RCK1	p.Phe502Val	1				GOF (Hinckley 2023, McTague 2018)
**RCK1**	**p.Met516Val**	**4**				GOF (Hinckley 2023, Rizzo 2016[40])
RCK1	p.Arg538Cys		1			
RCK1	p.Gln550del	1				GOF (Numis 2018[15])
	p.Lys629Asn	1				GOF (Hinckley 2023, Mikati 2015)
	p.Lys629Glu	2	1			GOF (Hinckley 2023)
	p.Gly652Val		1			LOF (Hinckley 2023)
	p.Asp718Val			1 (3*)		
	**p.Ile760Met**	**3**				GOF (Hinckley 2023)
	p.Ile760Phe	1				
	p.Leu781Val		1			
RCK2	p.Tyr796His			2 (5*)		LOF (Hinckley 2023)/ GOF (Milligan 2014, Mikati 2015)
**NADBD**	**p.Glu893Lys**	**3**				
NADBD	p.Glu893Val	1				
NADBD	p.Met896Ile	2	1	1		GOF (Milligan 2014)
NADBD	p.Met896Lys	1				GOF (Hinckley 2023, McTague 2018)
NADBD	p.Met896Val	1	1	1		
NADBD	p.Ala899Val	1				
NADBD	p.Arg905Gln		1			
NADBD	p.Gln906His		1			GOF (Hinckley 2023)
NADBD	p.Phe909Leu	1				
RCK2	p.Pro924Leu	1	1			GOF (Barcia 2012)
**RCK2**	**p.Arg928Cys**			**4 (13*)**	**2**	GOF (Hinckley 2023, Milligan 2014)
RCK2	p.Arg929Gln	1				

RCK2	p.Phe932Ile	0	1			LOF (Evely 2017[41])
RCK2	p.Phe932Leu	1		1		
RCK2	p.Phe932Ser	2				
RCK2	p.Arg933Cys			1 (2*)		
RCK2	p.Arg933Gly	1				
RCK2	p.Arg933His	1				
**RCK2**	**p.Ala934Thr**	**23**	**9**	**4**		GOF (McTague 2018, Milligan 2014)
RCK2	p.Ser937Gly				1	GOF (Mosca et al. 2024[35])
RCK2	p.Leu942Phe		0	1		
RCK2	p.Lys947Glu	1	1			
**RCK2**	**p.Arg950Gln**	**8**	**1**	**3 (6*)**	**2**	GOF (Hinckley 2023)
**RCK2**	**p.Arg961His**	**1**	**2**	**3**	**1 (3*)**	
RCK2	p.Arg961Ser	1				GOF (Hinckley 2023)
RCK2	p.Leu962Pro	1				
RCK2	p.Ala965Thr			1		
RCK2	p.Ala965Val	1				
**RCK2**	**p.Ala966Thr**			**3 (4*)**		GOF (Hinckley 2023)
RCK2	p.Ala966Thr (x2)		1			
RCK2	p.Ala989Ser	1				
Cter	p.Arg1106Gln				1	
Cter	p.Arg1106Pro	1				GOF (Hinckley 2023)
	Total général	180 (181*)	59 (59*)	36 (62*)	12 (14*)	

Supplementary Table 5: **Listing of reported KCNT1 variant and associated phenotypes**

One variant per family and phenotype in yellow

* In parentheses, the total number of patients reported, with each case counted, including familial cases.

Abbreviations: DEE: Developmental and Epileptic Encephalopathy, EIMFS: Epilepsy of Infancy with Migrating Focal Seizures, GOF: Gaon Of Function, LOF: Loss Of Function, SHE: Sleep-related Hypermotor Epilepsy

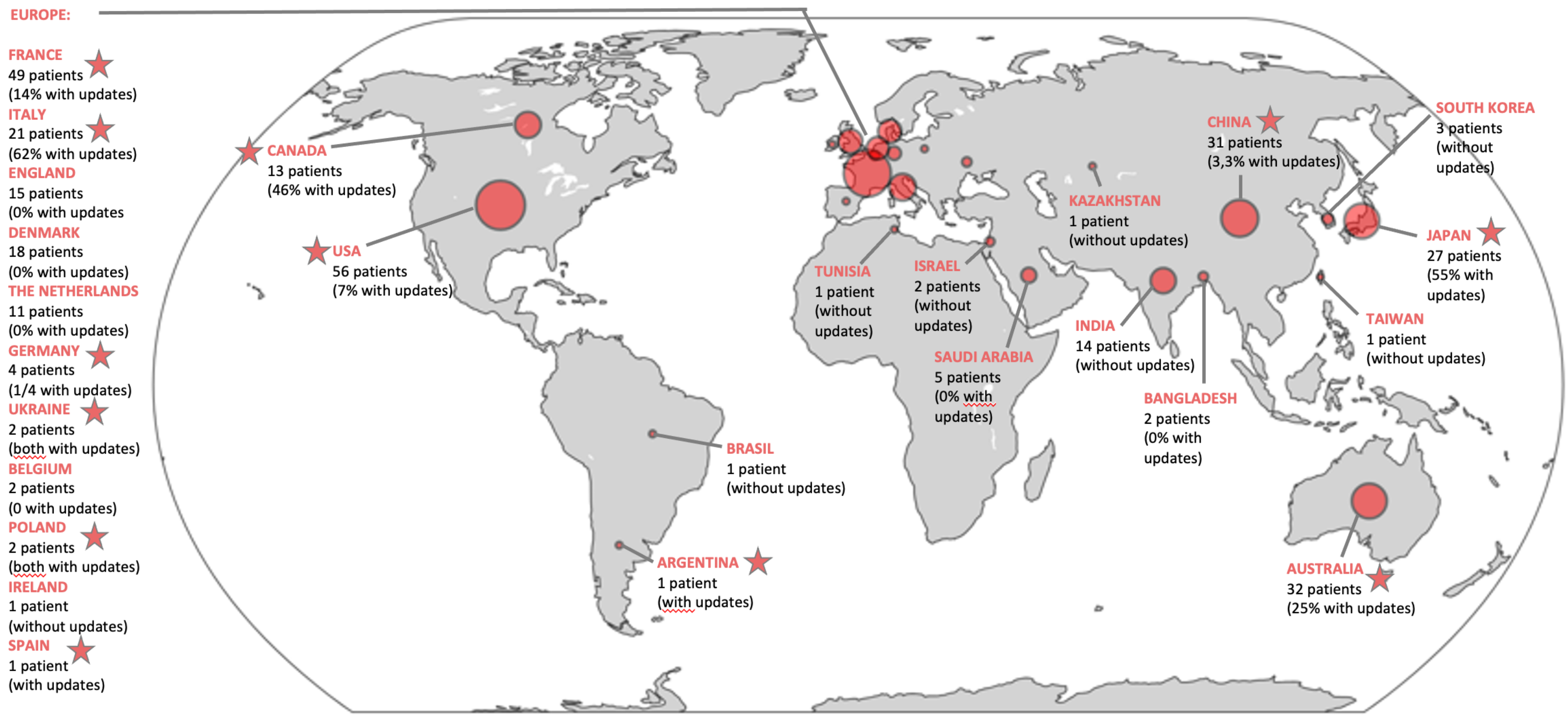

Supplementary Figure 1: Geographic distribution of published patients worldwide and response rate to the follow-up questionnaire

Bibliography:

1. Bonardi, C. M. *et al.* KCNT1-related epilepsies and epileptic encephalopathies: phenotypic and mutational spectrum. *Brain* 144, 3635–3650 (2021).
2. Fitzgerald, M. P. *et al.* Treatment Responsiveness in KCNT1-Related Epilepsy. *Neurotherapeutics* 16, 848–857 (2019).
3. Cherian, C. *et al.* The phenotypic spectrum of KCNT1: a new family with variable epilepsy syndromes including mild focal epilepsy. *J Neurol* 269, 2162–2171 (2022).
4. Dilena, R. *et al.* Early Treatment with Quinidine in 2 Patients with Epilepsy of Infancy with Migrating Focal Seizures (EIMFS) Due to Gain-of-Function KCNT1 Mutations: Functional Studies, Clinical Responses, and Critical Issues for Personalized Therapy. *Neurotherapeutics* 15, 1112–1126 (2018).
5. Ferretti, A. *et al.* Therapeutic Drug Monitoring of Quinidine in Pediatric Patients with KCNT1 Genetic Variants. *Pharmaceutics* 14, 2230 (2022).
6. Ikeda, A., Ueda, H., Matsui, K., Iai, M. & Goto, T. Recurrent pulmonary hemorrhage in juvenile patients with KCNT1 mutation. *Pediatrics International* 63, 352–354 (2021).
7. Ohba, C. *et al.* De novo KCNT1 mutations in early-onset epileptic encephalopathy. *Epilepsia* 56, e121-128 (2015).
8. Kawasaki, Y. *et al.* Three Cases of KCNT1 Mutations: Malignant Migrating Partial Seizures in Infancy with Massive Systemic to Pulmonary Collateral Arteries. *J Pediatr* 191, 270–274 (2017).
9. Kohli, U., Ravishankar, C. & Nordli, D. Cardiac phenotypic spectrum of KCNT1 mutations. *Cardiology in the Young* 30, 1935–1939 (2020).
10. Kravetz, M. C. *et al.* Case Report of Novel Genetic Variant in KCNT1 Channel and Pharmacological Treatment With Quinidine. Precision Medicine in Refractory Epilepsy. *Front Pharmacol* 12, 648519 (2021).
11. Kuchenbuch, M. *et al.* KCNT1 epilepsy with migrating focal seizures shows a temporal sequence with poor outcome, high mortality and SUDEP. *Brain* 142, 2996–3008 (2019).
12. Barcia, G. *et al.* De novo gain-of-function KCNT1 channel mutations cause malignant migrating partial seizures of infancy. *Nat Genet* 44, 1255–1259 (2012).
13. Kim, G. E. *et al.* Human slack potassium channel mutations increase positive cooperativity between individual channels. *Cell Rep* 9, 1661–1672 (2014).
14. Merdariu, D., Delanoë, C., Mahfoufi, N., Bellavoine, V. & Auvin, S. Malignant migrating partial seizures of infancy controlled by stiripentol and clonazepam. *Brain Dev* 35, 177–180 (2013).
15. Numis, A. L. *et al.* Lack of response to quinidine in KCNT1-related neonatal epilepsy. *Epilepsia* 59, 1889–1898 (2018).
16. Poisson, K., Wong, M., Lee, C. & Cilio, M. R. Response to cannabidiol in epilepsy of infancy with migrating focal seizures associated with KCNT1 mutations: An open-label, prospective, interventional study. *Eur J Paediatr Neurol* 25, 77–81 (2020).
17. Cornet, M.-C. *et al.* Neonatal presentation of genetic epilepsies: Early differentiation from acute provoked seizures. *Epilepsia* 62, 1907–1920 (2021).
18. Trivisano, M. *et al.* Fluoxetine Treatment in Epilepsy of Infancy with Migrating Focal Seizures Due to Variants: An Open Label Study. *Annals of Neurology* n/a,.
19. Yoshitomi, S. *et al.* Quinidine therapy and therapeutic drug monitoring in four patients with KCNT1 mutations. *Epileptic Disord* 21, 48–54 (2019).
20. Chong, P. F., Nakamura, R., Saitsu, H., Matsumoto, N. & Kira, R. Ineffective quinidine therapy in early onset epileptic encephalopathy with KCNT1 mutation. *Ann Neurol* 79, 502–503 (2016).

21. Datta, A. N., Michoulas, A., Guella, I., EPGEN Study & Demos, M. Two Patients With KCNT1-Related Epilepsy Responding to Phenobarbital and Potassium Bromide. *J Child Neurol* 34, 728–734 (2019).
22. Filareto, I. *et al.* Pharmacological approaches in drug-resistant pediatric epilepsies caused by pathogenic variants in potassium channel genes. *Front Cell Neurosci* 18, 1512365 (2024).
23. Fukuoka, M. *et al.* Quinidine therapy for West syndrome with KCNTI mutation: A case report. *Brain Dev* 39, 80–83 (2017).
24. Krygier, M. *et al.* Next-generation sequencing testing in children with epilepsy reveals novel clinical, diagnostic and therapeutic implications. *Front Genet* 14, 1300952 (2023).
25. Shchubelka, K. *et al.* Genetic determinants of global developmental delay and intellectual disability in Ukrainian children. *J Neurodev Disord* 16, 13 (2024).
26. Tsang, J. P. K. *et al.* Arginase deficiency with new phenotype and a novel mutation: contemporary summary. *Pediatr Neurol* 47, 263–269 (2012).
27. Vanderver, A. *et al.* Identification of a novel de novo p.Phe932Ile KCNT1 mutation in a patient with leukoencephalopathy and severe epilepsy. *Pediatr Neurol* 50, 112–114 (2014).
28. Yamamoto, K. *et al.* Synchronous heart rate reduction with suppression-burst pattern in KCNT1-related developmental and epileptic encephalopathies. *Epilepsia Open* 8, 651–658 (2023).
29. Derry, C. P. *et al.* Severe autosomal dominant nocturnal frontal lobe epilepsy associated with psychiatric disorders and intellectual disability. *Epilepsia* 49, 2125–2129 (2008).
30. Heron, S. E. *et al.* Missense mutations in the sodium-gated potassium channel gene KCNT1 cause severe autosomal dominant nocturnal frontal lobe epilepsy. *Nat Genet* 44, 1188–1190 (2012).
31. Mullen, S. A. *et al.* Precision therapy for epilepsy due to KCNT1 mutations: A randomized trial of oral quinidine. *Neurology* 90, e67–e72 (2018).
32. Hildebrand, M. S. *et al.* A targeted resequencing gene panel for focal epilepsy. *Neurology* 86, 1605–1612 (2016).
33. Licchetta, L. *et al.* Sleep-related hypermotor epilepsy (SHE): Contribution of known genes in 103 patients. *Seizure* 74, 60–64 (2020).
34. Cataldi, M. *et al.* Migrating focal seizures in Autosomal Dominant Sleep-related Hypermotor Epilepsy with KCNT1 mutation. *Seizure* 67, 57–60 (2019).
35. Mosca, I. *et al.* Case report: Marked electroclinical improvement by fluoxetine treatment in a patient with KCNT1-related drug-resistant focal epilepsy. *Front Cell Neurosci* 18, 1367838 (2024).
36. Hinckley, C. A. *et al.* Functional evaluation of epilepsy associated KCNT1 variants in multiple cellular systems reveals a predominant gain of function impact on channel properties. *Epilepsia* https://doi.org/10.1111/epi.17648 (2023) doi:10.1111/epi.17648.
37. McTague, A. *et al.* Migrating partial seizures of infancy: expansion of the electroclinical, radiological and pathological disease spectrum. *Brain* 136, 1578–1591 (2013).
38. Milligan, C. J. *et al.* KCNT1 gain of function in 2 epilepsy phenotypes is reversed by quinidine. *Ann Neurol* 75, 581–590 (2014).
39. Mikati, M. A. *et al.* Quinidine in the treatment of KCNT1-positive epilepsies. *Ann Neurol* 78, 995–999 (2015).
40. Rizzo, F. *et al.* Characterization of two de novoKCNT1 mutations in children with malignant migrating partial seizures in infancy. *Mol Cell Neurosci* 72, 54–63 (2016).
41. Evely, K. M., Pryce, K. D. & Bhattacharjee, A. The Phe932Ile mutation in KCNT1 channels associated with severe epilepsy, delayed myelination and leukoencephalopathy produces a loss-of-function channel phenotype. *Neuroscience* 351, 65–70 (2017).
